# Depletion of Fat Mass and Obesity‐Associated Protein (FTO) Drives Heterochromatin Loss via Lysine Acetyltransferase 8 (KAT8)‐Mediated Remodeling and Spacing Factor 1 (RSF1) Acetylation in Skin Aging

**DOI:** 10.1002/mco2.70205

**Published:** 2025-07-09

**Authors:** Fan Wang, Lei Zhou, Yun Zhong, Yisheng Cai, Xin Meng, Mengting Chen, Rui Mao, Xin Xiao, Caitan Yi, Yi Guo, Hongfu Xie, Yiya Zhang, Ji Li

**Affiliations:** ^1^ Department of Dermatology, Xiangya Hospital Central South University Changsha China; ^2^ Hunan Key Laboratory of Aging Biology, Xiangya Hospital Central South University Changsha China; ^3^ National Clinical Research Center for Geriatric Disorders, Xiangya Hospital Central South University Changsha China; ^4^ Department of Dermatology, the Third Affiliated Hospital Sun Yat‐sen University Guangzhou China; ^5^ Department of Dermatology, Nanfang Hospital Southern Medical University Guangzhou China

**Keywords:** fat mass and obesity‐associated protein, lysine acetyltransferase 8, N6‐methyladenosine, remodeling and spacing factor 1, senescence

## Abstract

N6‐methyladenosine (m6A), as the most common RNA modification at the post‐transcriptional level, plays a role in various pathophysiological processes. However, its underlying mechanism in skin aging remains enigmatic. Here, we identified that fat mass and obesity‐associated protein (FTO) serves as a protective factor against skin aging. FTO expression is downregulated in aging skin tissues and senescent fibroblasts. Furthermore, the depletion or inhibition of FTO exacerbates dermal fibroblasts senescence and accelerates skin aging. Additionally, RNA‐seq combined with MeRIP‐seq revealed that lysine acetyltransferase 8 (KAT8) is the downstream target of FTO. FTO deficiency leads to an increase in m6A levels and a decrease in mRNA stability of KAT8 in a m6A‐YTHDF2‐dependent manner. Notably, our integrated analysis of m6A sequencing and acetylation proteomics links changes in heterochromatin structure with aging. Mechanistically, KAT8 depletion leads to heterochromatin loss and the subsequent aging by acetylating remodeling and spacing factor 1 (RSF1) at K1050. Overall, our finding reveals a pivotal role of FTO‐mediated m6A modification in the skin aging by regulating KAT8/RSF1‐involved heterochromatin formation. It provides new insights into the mechanisms and strategies for delaying aging and improving healthspan.

## Graphical abstract

FTO deficiency leads to an increase in m6A levels and a decrease in mRNA stability of KAT8. Subsequently, KAT8 depletion leads to heterochromatin loss and the subsequent aging by acetylating RSF1 at K1050. Overall, the FTO–KAT8–RSF1 axis is a new target for delaying skin aging.

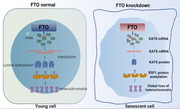

## 1 Introduction

The skin is the largest organ of the human body and serves as the interface between the internal and external environments. It plays a vital role in barrier protection and directly reflects the health status of the body [1, 2]. Skin aging is characterized by thinning, fine lines and relaxation of the skin, along with a general decline in resistance to external insults, infection and regenerative capacity. Recent studies demonstrated that the accumulation of senescent cells is the major driver of aging and age‐related disease [3, 4]. A large number of senescent fibroblasts accumulate in aging skin, leading to alterations in both the structure and function of the skin [5, 6].

Senescent cells undergo marked characteristic phenotypic changes, including cell cycle arrest and the accumulation of senescence‐associated β‐galactosidase (SA‐β‐Gal) [7]. A global loss of heterochromatin is a hallmark of aging in eukaryotes and is considered a contributing factor to the aging process [8, 9, 10]. Moreover, declines of heterochromatin markers, such as heterochromatinprotein1a (HP1α), have been reported to be associated with age‐related impairment. Notably, heterochromatin restoration has been shown to extend the lifespan of flies [8, 9, 11].

N6‐methyladenosine (m6A) modification, the methylation of an adenosine base at the nitrogen‐6 position of an mRNA, has garnered increasing attention for its crucial role in various biological processes, including aging [12]. It is dynamically reversible and is mainly regulated by methyltransferases (WTAP, METTL3, and METTL14) [13, 14], demethylases (FTO and ALKBH5) [15, 16], and RNA‐binding proteins (YTH domain family proteins, heterogeneous nuclear ribonucleoprotein (HNRP) family and the insulin‐like growth factor 2 mRNA‐binding protein family). Among them, methyltransferases and demethyltransferases are responsible for the m6A modification of targets, while RNA‐binding proteins are recruited by m6A, which affect the expression of target genes by regulating mRNA nucleation, degradation, selective splicing and translation, and ultimately participate in many biological processes. Our previous work demonstrated the role of WTAP‐decreased m6A levels in senescent fibroblasts [17]. Nevertheless, the link between m6A modification and heterochromatin loss requires further exploration.

Fat mass and obesity‐associated protein (FTO) is the first identified m6A demethylase shown to exhibit m6A demethylation activity on mRNA. Previous studies have demonstrated that FTO depletion induced G1 cell cycle arrest by downregulating cyclin D1 expression in an m6A‐dependent manner [18]. Additionally, FTO was reported to be reduced in human‐aged ovarian granulosa cells (GCs), and FTO knockdown accelerated GCs senescence [19]. Moreover, recent studies have found that IL‐17A can upregulate the FTO expression by activating the JNK signal pathway to promote endothelial cell senescence [20]. These reports suggest that FTO may be involved in various cell senescence; however, its specific function in skin aging remains poorly understood.

Here, we report that FTO serves as a key protective factor against skin aging. Mechanistically, the deletion of FTO leads to a decrease in KAT8 expression in an m6A‐dependent manner, which subsequently results in reduced acetylation of RSF1 and the subsequent age‐related heterochromatin loss. These findings underscore the significant potential of FTO as a novel target for mitigating skin aging.

## 2 Results

### 2.1 FTO Is Downregulated in Aging Skin Tissues and Senescent Fibroblasts

In an attempt to screen the m6A modification that are related to skin aging, we analyzed the expression profiles of 16 m6A‐related genes in human dermal fibroblasts (HDFs) from young individuals and those with premature senility syndrome using GEO dataset (GSE113957). Among them, the expression of FTO was downregulated considerably in individuals with premature senility syndrome (Figure S1A,B). Given the pivotal role of m6A demethyltransferase, particularly FTO, in a variety of biological processes, FTO was selected for additional study.

**FIGURE 1:**
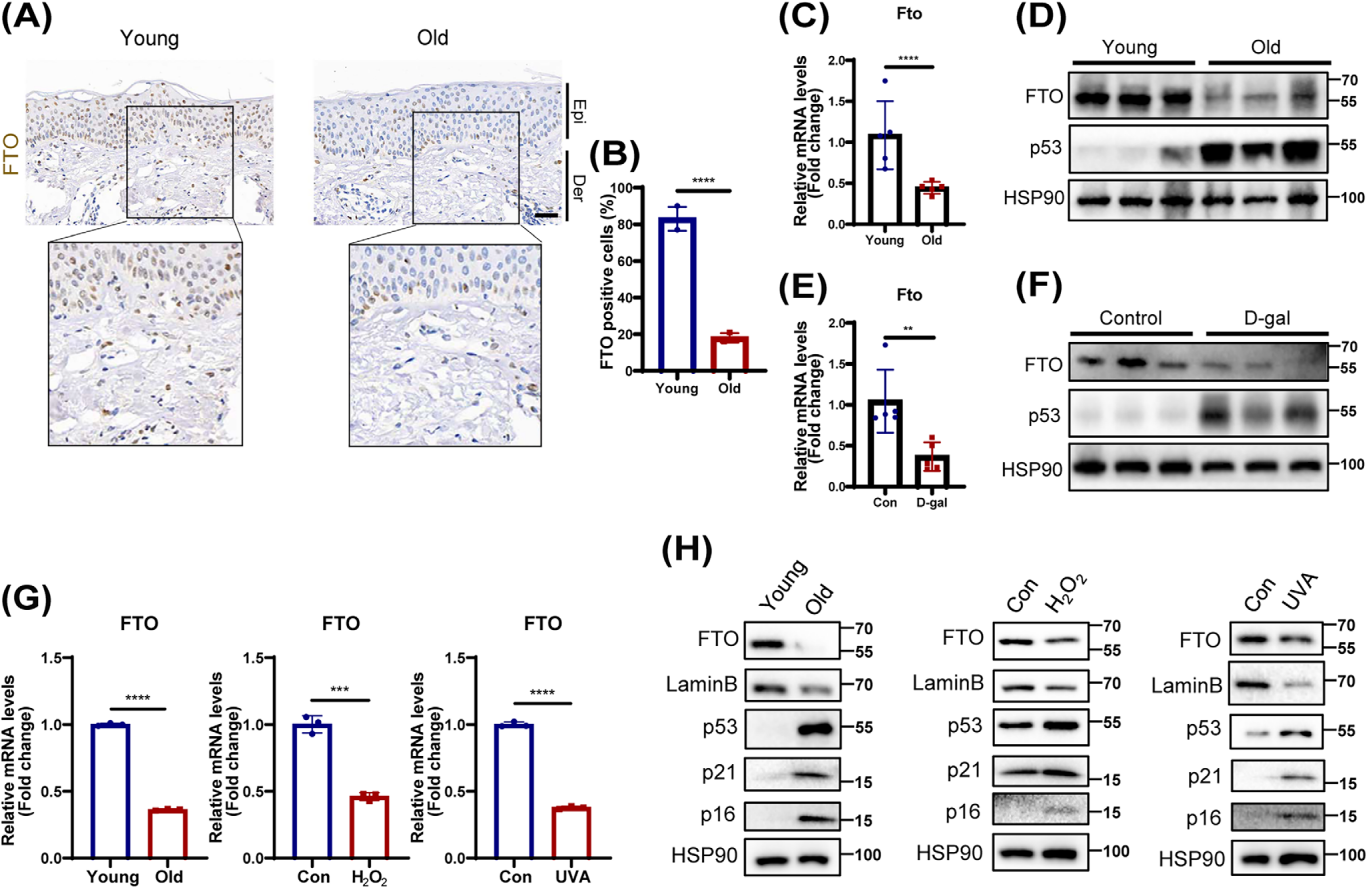
Fat mass and obesity‐associated protein (FTO) expression was downregulated in aging skin tissues and different senescent human dermal fibroblasts (HDFs) models. (A and B) Immunohistochemical staining (A) in human skin of young people (*n* = 6, mean age: 23.5) and the elderly (*n* = 6, mean age: 76.5), and the staining density was quantified by ImageJ (B). (C–F) Quantitative PCR (qPCR) assay and western blot detection revealed the expression of FTO in the skin of natural aging mice (*n* = 5; aged mice: 20‐month‐old mice, young mice: 2‐month‐old mice) (C and D) and D‐gal‐induced premature senile mice (*n* = 5) (E and F), respectively. (G and H) The expression changes of mRNA (G) and protein (H) levels of FTO in different senescent HDFs models. Data are representative of at least three independent experiments. Scale bar, 50 µm. The data showed an average of ± SEM. ns, not significant; *p < 0.05; **p < 0.01; ***p < 0.001; ****p < 0.0001.

Next, we verified the FTO expression in the skin tissues from the young and elderly. Consistent with the results of GEO, a significantly lower expression of FTO was detected in aging human skin tissues by immunochemical analysis (Figure 1A,B). Similarly, a marked decline in FTO expression was observed in the skin of naturally aged mice (Figure 1C,D) and D‐galactose (D‐gal)‐induced aging mice (Figure 1E,F). Interestingly, single‐cell RNA sequencing data and immunofluorescence analysis of human skin tissues revealed that FTO was primarily located in fibroblasts (Figures S2 and S3). Moreover, the FTO expression in fibroblasts was significantly reduced with aging (Figure S3). Given that senescent fibroblasts are known to contribute substantially to skin aging [21], we then further assessed FTO expression in three models of cell senescence using HDFs. As expected, FTO expression was also decreased in the replicative senescence, as well as in UVA‐ and hydrogen peroxide (H_2_O_2_)‐induced senescent HDFs (Figure 1 G,H). Based on the above results, FTO was lowly expressed in aging skin tissues and senescent HDFs, potentially implicating it in the progression of aging.

### 2.2 FTO Protects HDFs Against Senescence

**FIGURE 2:**
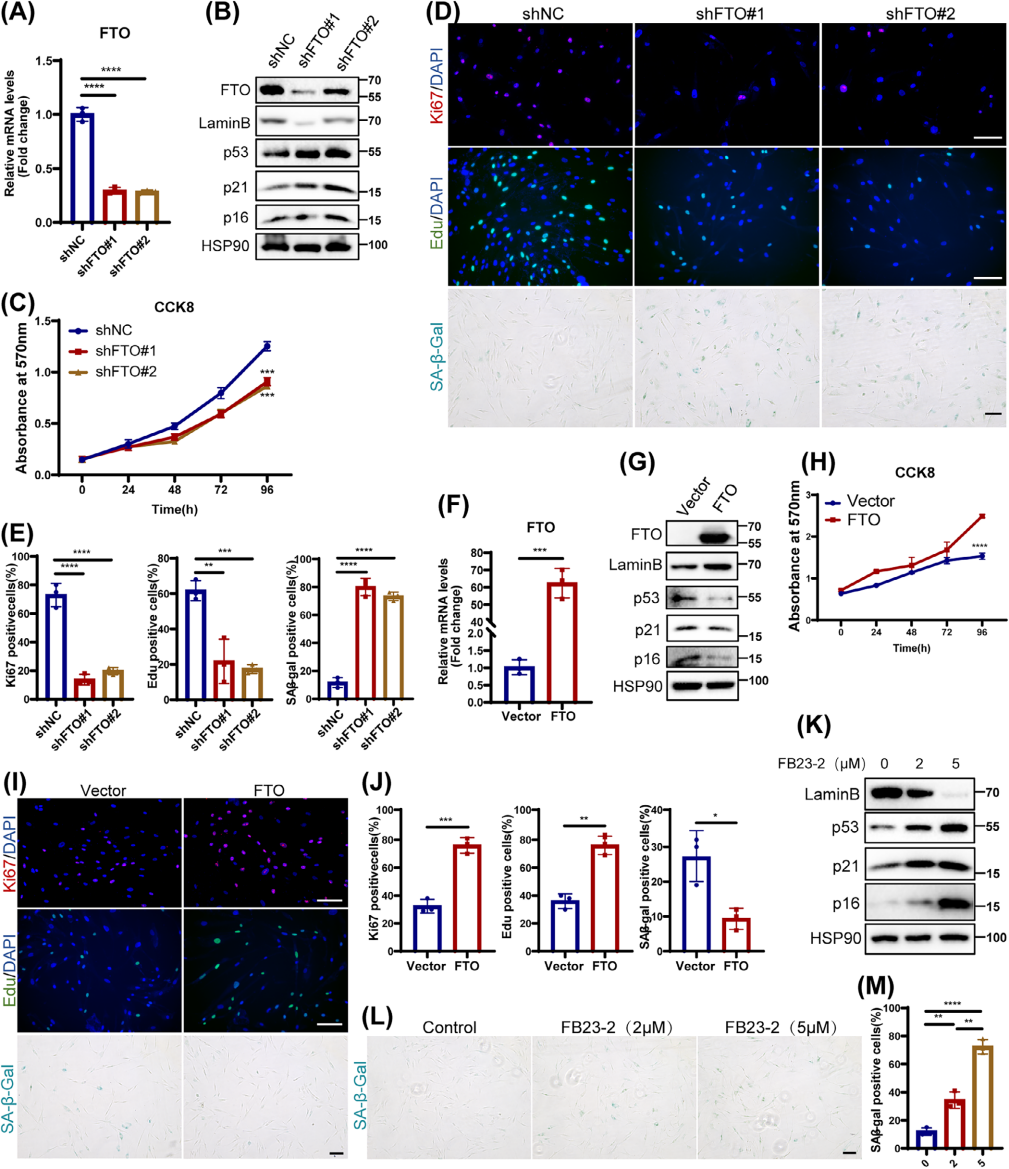
Expression of fat mass and obesity‐associated protein (FTO) was related to senescent phenotype of human dermal fibroblasts (HDFs). Young HDFs (PD < 10) were infected with shFTO or shNC lentivirus. (A) The expression of FTO was analyzed by RT‐quantitative PCR (qPCR). (B) The protein levels of FTO, Lamin B, p16, p21, and p53 by western blotting. (C) CCK8 assay. (D) Immunofluorescence staining of Ki67 and EdU, senescence‐associated β‐galactosidase (SA‐β‐Gal) staining in HDFs, and positive staining cells were quantified with ImageJ (E). Senescent HDFs (PD > 40) were transfected with FTO or Vector lentivirus. (F) The expression of FTO was analyzed by RT‐qPCR. (G) The protein levels of FTO, Lamin B, p16, p21, and p53 by western blotting. (H) CCK8 assay. (I) Immunofluorescence staining of Ki67, EdU, and SA‐β‐Gal staining in HDFs upon ectopic of FTO, and the percentage of positive staining cells were quantified (J). (K) After treatment with different concentrations of FB23‐2 (0 µM, 2 µM, and 5 µM), the protein level of Lamin B, p16, p21, and p53. (L) SA‐β‐Gal staining in HDFs treated with FB23‐2, and statistical analysis (M). Data are representative of at least three independent experiments. Scale bar, 100 µm. ns, not significant; *p < 0.05; **p < 0.01; ***p < 0.001; ****p < 0.0001.

To investigate the potential role of FTO in cellular senescence, we knocked down FTO in young HDFs using two independent short hairpins RNAs (shRNAs). Efficiency of FTO knockdown in HDFs was confirmed by quantitative PCR (qPCR) and western blotting (Figure 2A,B). Subsequently, we found that depletion of FTO induced hallmark features of cellular senescence, including increased expression of cell cycle‐related senescence indexes (p16, p21, and p53), reduced Lamin B levels (Figure 2B), and repressed cell proliferation (Figure 2C–E). Moreover, a significant increase in SA‐β‐Gal staining (Figure 2D,E) and elevated levels of senescence‐associated secretory phenotype (SASP; IL‐1β, IL‐6, and IL‐8; Figure S8A), were also observed in HDFs with FTO knockdown. It was worth mentioning that FTO deficiency induced senescence rather than apoptosis using flow cytometric analysis (Figure S4A,B). Conversely, FTO overexpression in senescent HDFs showed the opposite effects (Figures 2F–J and S8B).

Next, we utilized FB23‐2, a selective chemical inhibitor of FTO to further verify the role of FTO in HDFs senescence. A significant senescent phenotype was observed in HDFs following treatment with FB23‐2, marked by elevated protein levels of p16, p21, and p53, as well as a reduced level of Lamin B (Figure 2K), and an observably increase of SA‐β‐Gal staining (Figure 2L,M). Notably, low doses of FB23‐2 treatment preferentially induced cellular senescence over apoptosis, as confirmed by flow cytometry (Figure S4C,D). The above results were similar to the effects of FTO deficiency, implicating that FTO inhibition could also promote HDFs senescence. In conclusion, these results strongly demonstrate a protective role of FTO in cellular senescence.

### 2.3 FTO Targets and Stabilizes KAT8 via an m6A‐YTHDF2‐Dependent Pathway

**FIGURE 3:**
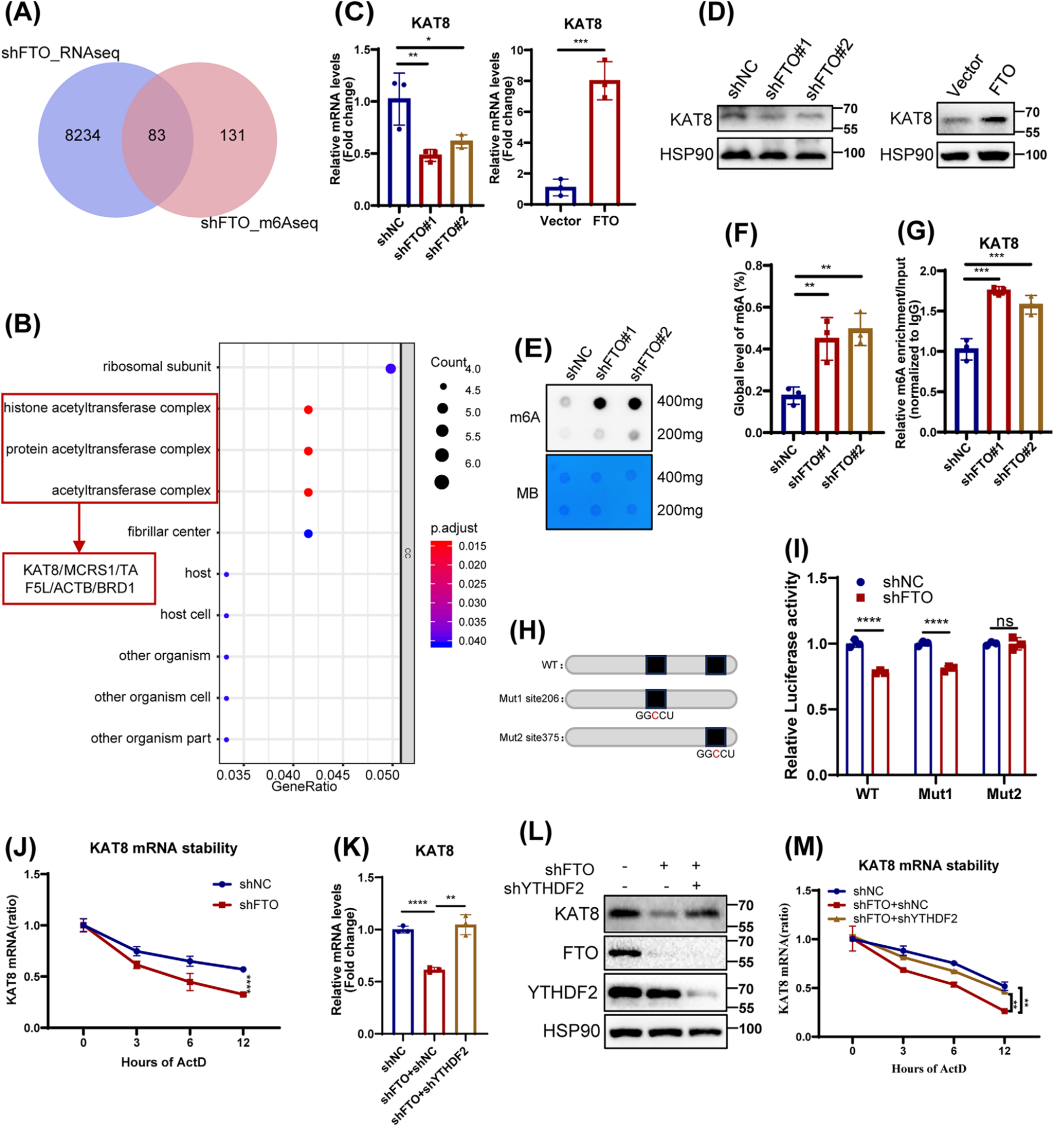
Lysine acetyltransferase 8 (KAT8) expression was regulated by fat mass and obesity‐associated protein (FTO) in m6A‐dependent manner. (A) The Venn diagram was generated by the DEGs of RNA‐seq from FTO‐depleted human dermal fibroblasts (HDFs) and the of m6A‐seq in GSE124509. (B) Gene ontology (GO) analysis of overlapping genes. (C and D) KAT8 expression was evaluated by quantitative PCR (qPCR) (C) and western blotting (D) in FTO‐silenced or overexpressed HDFs. (E and F) Dot blot (E) and m6A colorimetric assay (F) showed the overall m6A modification in FTO‐knockdown HDFs; the loading control was methylene blue staining. (G) MeRIP analysis combined with qPCR was employed to detect the m6A modification of KAT8 in FTO‐depleted HDFs. The relative m6A enrichment in each group was calculated by m6A‐IP/input and IgG‐IP/input. (H) The 401 bp‐length of KAT8 mRNA 3′UTR sequence was analyzed by JASPAR web tool to predict the possible FTO binding sites. (I) The wild‐type (full‐length) or mutant KAT8 mRNA 3′UTR plasmids were cotransfected with shFTO or shNC, respectively, and relative luciferase activities were detected. (J) qPCR analysis of the mRNA stability of KAT8 in HDFs with or without FTO knockdown. (K and L) qPCR and western blot analysis of the mRNA (K) and protein (L) levels of KAT8 in FTO‐knockdown HDFs, in combination with knockdown of the m6A reader YTH N6‐methyladenosine RNA‐binding protein 2 (YTHDF2). (M) qPCR analysis of the mRNA stability of KAT8. Data are representative of at least three independent experiments. Data are shown as mean ± SEM. ns, not significant; *p < 0.05; **p < 0.01; ***p < 0.001; ****p < 0.0001.

Next, RNA‐seq was used to further elucidate the potential mechanism by which FTO depletion induced HDFs senescence. Analysis revealed 2855 downregulated and 5599 upregulated differentially expressed genes (DEGs) in FTO‐deficiency HDFs (Figure S5A,B). Gene ontology (GO) analysis showed that DEGs were primarily enriched in catalytic activity and extracellular matrix formation pathways (Figure S5C), while KEGG analysis highlighted their involvement in Ras signaling, calcium signaling, and other related pathways (Figure S5D). To explore direct FTO targets modulated via m6A modification, we next combined the DEGs in RNA‐seq with the potential FTO targets identified by MeRIP‐seq (GSE124509), identifying 83 overlapping genes (Figure 3A). GO analysis of these overlapping genes revealed significant enrichment in the acetylase complex pathway (Figure 3B), consistent with previous reports linking acetylation modifications to aging processes [22, 23]. Subsequently, we employed qPCR to examine the expression of acetylase‐related overlapping genes (KAT8, MCRS1, TAF5L, ACTB, and BRD1) in FTO‐deficient HDFs (Figures 3C and S5E). In line with the RNA‐seq results, the expression of KAT8, MCRS1, and TAF5L was reduced, while the expression of ACTB and BRD1 was upregulated in HDFs following FTO silencing. Notably, KAT8, as a critical acetyltransferase, exhibited the strongest correlation with the acetylation pathway. Consequently, KAT8 has been the focus of subsequent research.

Lysine acetyltransferase 8 (KAT8), a member of the MYST protein family, is highly conserved across species and involved in various biological processes by regulating acetylation levels of target protein [24, 25, 26]. Here, we found that both the mRNA and protein expression of KAT8 were downregulated in three senescent HDFs models (Figure S7A), and that FTO positively regulates KAT8 expression (Figure 3C,D). Considering that FTO as an important m6A demethyltransferase, we then analyzed the m6A levels in FTO‐deficiency HDFs by m6A dot blotting and RNA methylation quantitative determination. As expected, FTO knockdown resulted in a significant increase in total m6A levels (Figure 3E,F). Additionally, MeRIP‐qPCR assay showed an increase in m6A levels at the KAT8 mRNA following FTO depletion in HDFs (Figure 3G). Of note, MeRIP‐seq analysis further revealed that the m6A peaks were highly enriched in CDS and 3′UTR region of KAT8 mRNA (Figure S6A,B). Analysis of the m6A peak motif using exomePeak, MACS2, and MeTPeaK identified a conserved sequence, primarily GGACU (Figure S6C). Using SRAMP, we predicted two potential m6A modification sites within the 3′UTR of KAT8 (Figure S6D). Next, luciferase reporters containing either the wild type (WT) or mutant (Mut) of KAT8 mRNA 3′UTR sequence were constructed to explore the effect of m6A modification on KAT8 expression. For Mut reporters, the adenosine base (A) at the m6A site was replaced with cytosine (C) to eliminate the m6A methylation effect (Figure 3H). FTO knockdown decreased the luciferase activity of WT and Mut1 KAT8, but not Mut2 KAT8 (Figure 3I). Therefore, these results indicated that FTO regulated KAT8 expression in an m6A‐dependent pattern at site 375.

The m6A modification targets gene expression by influencing the alternative splicing, nucleation, stability, and translation of RNA. We noticed that the half‐life of KAT8 transcript was shortened in FTO‐depleted HDFs, as assessed by RNA stability assays (Figure 3J). YTH m6A RNA‐binding protein 2 (YTHDF2), a key m6A reader of target gene 3′UTR sites, was reported to selectively recognize and degrade m6A‐modified mRNA [27]. Given that YTHDF2 targets multiple transcripts to reduce their half‐life, we detected its role in regulating of KAT8 mRNA stability. Indeed, silencing YTHDF2 in FTO‐deficient HDFs resulted in upregulation of both KAT8 mRNA and protein levels (Figure 3K,L), in parallel with enhanced KAT8 mRNA stability (Figure 3M). Thus, our findings indicated that FTO upregulated KAT8 expression partly in a YTHDF2‐dependent manner.

### 2.4 KAT8 is Essential for FTO‐Regulated HDFs Senescence

**FIGURE 4:**
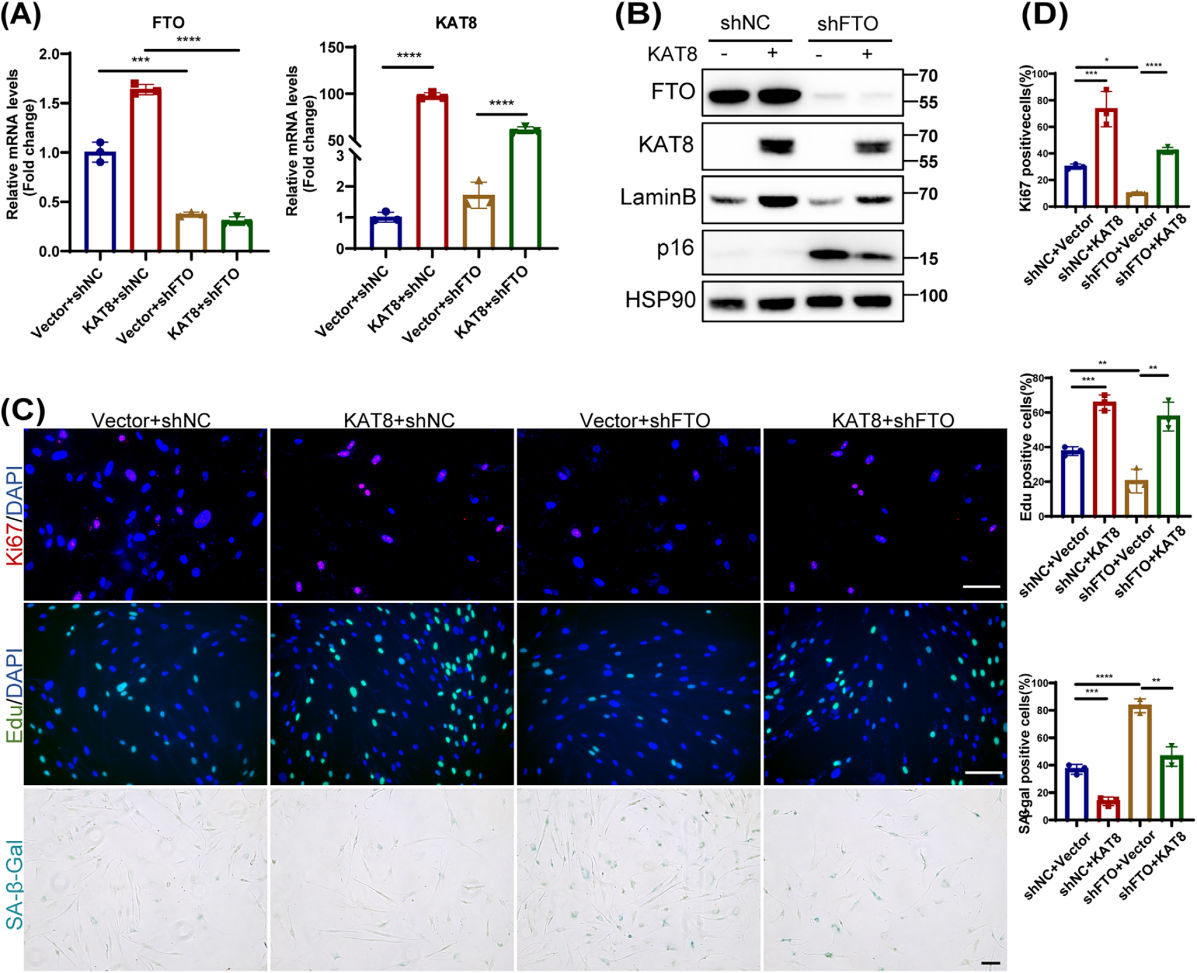
Lysine acetyltransferase 8 (KAT8) was involved in fat mass and obesity‐associated protein (FTO)‐mediated cellular senescence in human dermal fibroblasts (HDFs). (A) The mRNA level of FTO and KAT8 in FTO‐deprived HDFs following KAT8‐lentivirus transfection. (B) Expression of p53, p21, and p16 were verified in protein level. (C) Immunofluorescence staining of Ki67, EdU, and senescence‐associated β‐galactosidase (SA‐β‐Gal) staining were conducted. (D) represents the statistical results of (C). Data are representative of at least three independent experiments. Scale bar, 100 µm. Data are shown as mean ± SEM. ns, not significant; *p < 0.05; **p < 0.01; ***p < 0.001; ****p < 0.0001.

To reveal the role of KAT8 in FTO‐regulated HDFs senescence, we next knocked down or overexpressed KAT8 in young or old HDFs, and assessed various senescence markers. We found that the knockdown or overexpression of KAT8 significantly induced or delayed the senescent phenotype of HDFs, respectively (Figures S7 and S8C,D). Next, we overexpressed KAT8 in FTO‐deprived HDFs (Figure 4A,B), and the results showed that the ectopic expression of KAT8 reversed FTO depletion‐induced HDFs senescence, as evidenced by decreased p16 protein levels, reduced SA‐β‐Gal staining, attenuated SASP, and increased EdU incorporation, Ki67 immunoreactivity and Lamin B levels (Figures 4B–D and S8E). In summary, these results suggest that the protective role of FTO against HDFs senescence occurred mainly by KAT8‐dependent manner.

### 2.5 KAT8 Acetylates RSF1 at K1050 Residue

**FIGURE 5:**
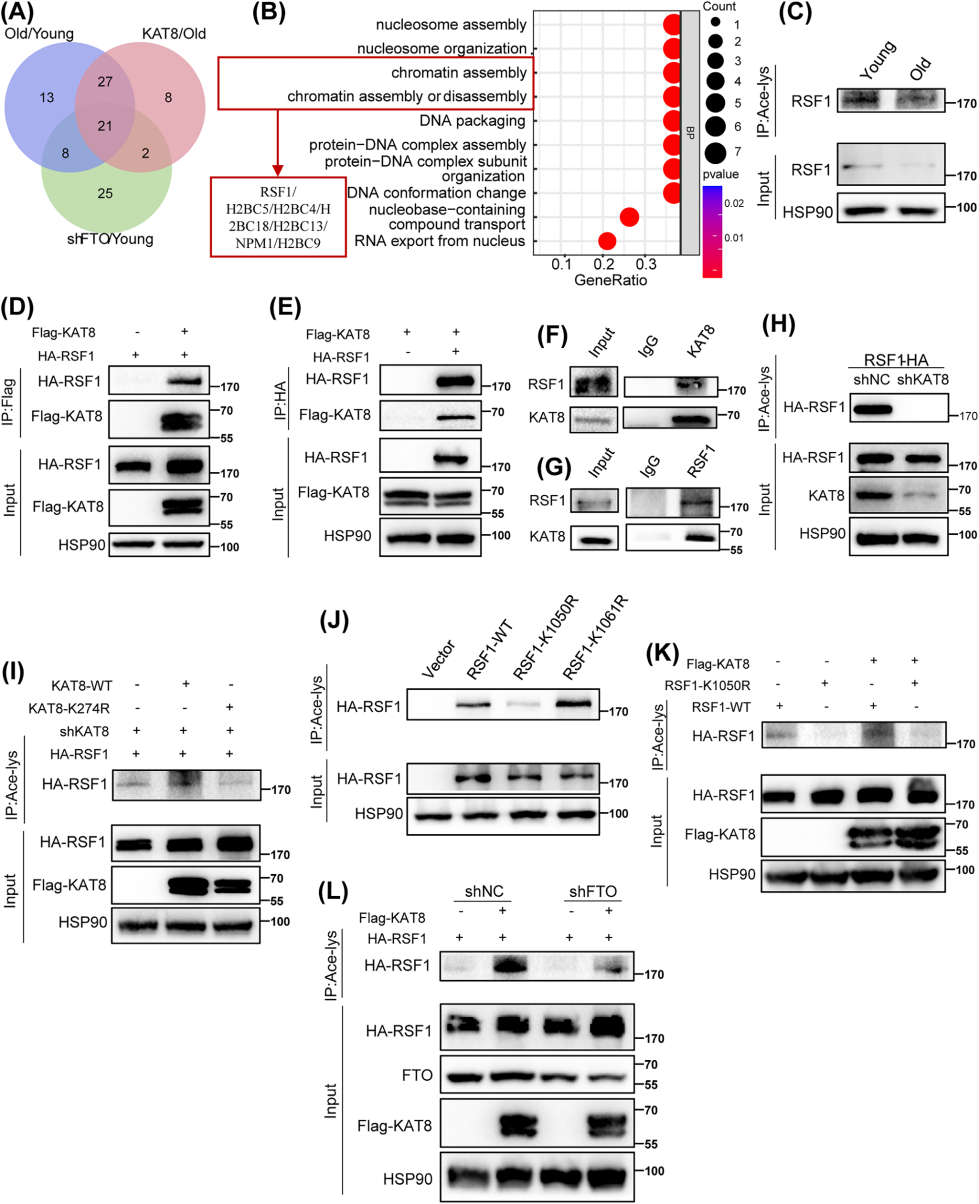
Acetylation at the K1050 site of RSF1 is regulated by lysine acetyltransferase 8 (KAT8). (A) The Venn diagram is generated from three sets of comparable MS data. (B) Gene ontology (GO) analysis of overlapping differentially expressed genes (DEGs) in (A). (C) Acetylation of remodeling and spacing factor 1 (RSF1) in young and old human dermal fibroblasts (HDFs). (D and E) Coimmunoprecipitation (CoIP) between ectopic Flag‐KAT8 and HA‐RSF1 in HEK293T cells. (F and G) CoIP between endogenous KAT8 and RSF1 in HDFs. (H) HEK293T exogenously expressing RSF1 were treated with shNC or shKAT8 lentivirus, and acetylation of HA‐RSF1 was detected. (I) CoIP of KAT8 mutants with HA‐RSF1 in HEK293T cells after endogenous KAT8 knockdown. (J) Acetylation of exogenous HA‐RSF1 WT, K1050R, and K1061R in HEK293 cells. (K) CoIP of Flag‐KAT8 with HA‐RSF1 WT or K1050R in HEK293T cells. (L) Acetylation of RSF1 in rescue assay in HEK293T cells. Data are representative of at least three independent experiments.

As an important acetyltransferase, KAT8 is involved in various biological processes, including chromatin structure regulation and cell lifespan by catalyzing H4K16 acetylation in mammalian cells [28]. To determine the substrate responsible for KAT8‐mediated HDFs senescence, we performed acetylated proteome by liquid chromatography–tandem mass spectrometry (LC–MS/MS) on young, old, FTO‐knockdown and KAT8‐overexpression HDFs. In total, we identified 497 acetylation sites across 300 proteins. We next evaluated and compared differential acetylated proteins and differential acetylation sites among the above four groups. A total of 69 differential proteins (111 differential acetylation sites) and 58 differential proteins (88 differential acetylation sites) were detected in the elderly group and FTO knockdown group, respectively, compared to the young group. In addition, 56 differential proteins (86 differential acetylation sites) were detected in the KAT8 overexpression group compared to the elderly group. Further analysis of the acetylated proteins in these three groups revealed 21 fully overlapping proteins (Figure 5A and Table S3). GO analysis revealed that the overlapping proteins were enriched in “chromatin assembly” pathway (Figure 5B).

RSF1, a component of the ATP‐dependent chromatin remodeling complex, plays a crucial role in chromatin remodeling [29, 30]. Since chromatin remodeling is a key epigenetic modifications and an important marker of aging [31], we hypothesized that RSF1 could be a target of KAT8, participating in cell senescence through the regulation of chromatin remodeling. First, using pan acetyl lysine antibodies, we observed a significant decrease in the acetylation level of RSF1 in senescent HDFs (Figure 5C). Next, coimmunoprecipitation (CoIP) analysis manifested the interaction between KAT8 and RSF1 both exogenously (Figure 5D,E) and endogenously (Figure 5F,G). Moreover, KAT8 silencing significantly reduced the acetylation level of RSF1 (Figure 5H). K274 residue was recognized to be the major site in the acetylation function of KAT8 [32]. To further validate role of KAT8 in RSF1 acetylation, shRNA targeting KAT8 3′UTR was designed to specifically knockdown the endogenous rather than exogenous KAT8 expression. Of note, the acetylation level of exogenous RSF1 was increased by KAT8‐WT but not KAT8‐K274R (a catalytic inactive mutant of KAT8; Figure 5I). We next investigated the sites on RSF1 that were acetylated by KAT8. Two potential acetylation sites, K1050 and K1061 of RSF1, were detected by acetylated proteomics, which were highly conserved across species. Following, we mutated this two lysine (K) to arginine (R), respectively, which mimics the deacetylation state of RSF1. It was found that RSF1‐K1050R but not RSF1‐K1061R showed significantly decreased acetylation, comparing to RSF1‐WT (Figure 5J). Consistently, forced expression of KAT8 further increased the acetylation of RSF1‐WT but not RSF1‐K1050R (Figure 5K). Moreover, FTO depletion significantly reduced the acetylation of RSF1, an effect that was partially reversed by KAT8 overexpression (Figure 5L). Collectively, these results indicated that K1050 was likely the major acetylation site in RSF1 regulated by KAT8.

### 2.6 The Declined RSF1 Acetylation Level Accounts for FTO/KAT8‐Depletion‐Induced Senescence

**FIGURE 6:**
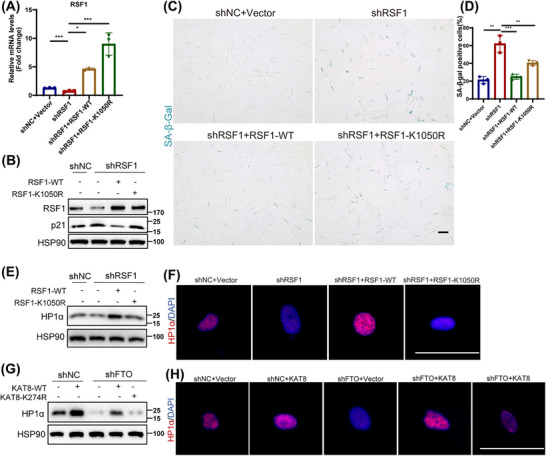
Remodeling and spacing factor 1 (RSF1; K1050) is essential for human dermal fibroblasts (HDFs) senescence. (A) The mRNA level of RSF1 in shRSF1 HDFs transfected with RSF1‐WT or RSF1‐K1050R lentivirus. (B) Expression of RSF1 and p21 in protein level. (C and D) Senescence‐associated β‐galactosidase (SA‐β‐Gal) staining (C) and the percentage of positive staining cells (D). (E and F) The expression of HP1α by western blotting (E) and immunofluorescence staining (F) after transfected with RSF1‐WT or RSF1‐K1050R lentivirus in shRSF1 HDFs. (G and H) The expression of heterochromatinprotein1a (HP1α) by western blotting (G) and immunofluorescence staining (H) after transfected with lysine acetyltransferase 8 (KAT8)‐WT or KAT8‐K274R lentivirus in shFTO HDFs. Data are representative of at least three independent experiments.

Given the established regulatory role of KAT8 on RSF1 acetylation, we further explored the impact of RSF1 acetylation in HDFs senescence. We designed two different shRNA targeting 3′UTR of RSF1 to silence RSF1 in HDFs. The results indicated that the stable knockdown of RSF1 promoted HDFs senescence (Figure S9). Next, we overexpressed either RSF1‐WT or RSF‐K1050R in shRSF1‐treated HDFs (Figure 6A,B). Importantly, RSF1‐WT, but not RSF1‐K1050R, alleviated the upregulation of P21 (Figure 6B), and the positive rate of SA‐β‐Gal staining (Figure 6C,D) induced by shRSF1. This indicated that acetylation of RSF1 at the K1050 site is the key mechanism by which RSF1 delayed HDFs senescence. Alejandra Loyola, et al. highlighted that RSF1 was an important regulator of chromatin remodeling [29, 30]. Whereafter, through western blot analysis and immunofluorescence analysis, we found that the loss of RSF1 decreased the expression of heterochromatin index HP1α (Figure 6E,F). Consistently, HP1α levels was upregulated in HDFs with overexpressed RSF1‐WT but not RSF1‐K1050R (Figure 6E,F). Furthermore, knockdown of FTO or KAT8 decreased HP1α levels, while overexpression of FTO or KAT8 increased it (Figure S10A–D). To explore whether FTO affect HP1α expression through KAT8‐mediated acetylation regulation, we examined HP1α levels in FTO‐depleted HDFs overexpressing either KAT8‐WT or the catalytic mutant KAT8‐K274R. As expected, FTO deficiency led to reduced HP1α expression, which was rescued by overexpressed KAT8‐WT but not KAT8‐K274R (Figure 6G,H). Collectively, these findings indicated that FTO/KAT8 deficiency induced cellular senescence by disrupting chromatin remodeling via affecting RSF1 acetylation.

### 2.7 FTO Elimination Leads to the Skin Aging Phenotypes in Mouse

**FIGURE 7:**
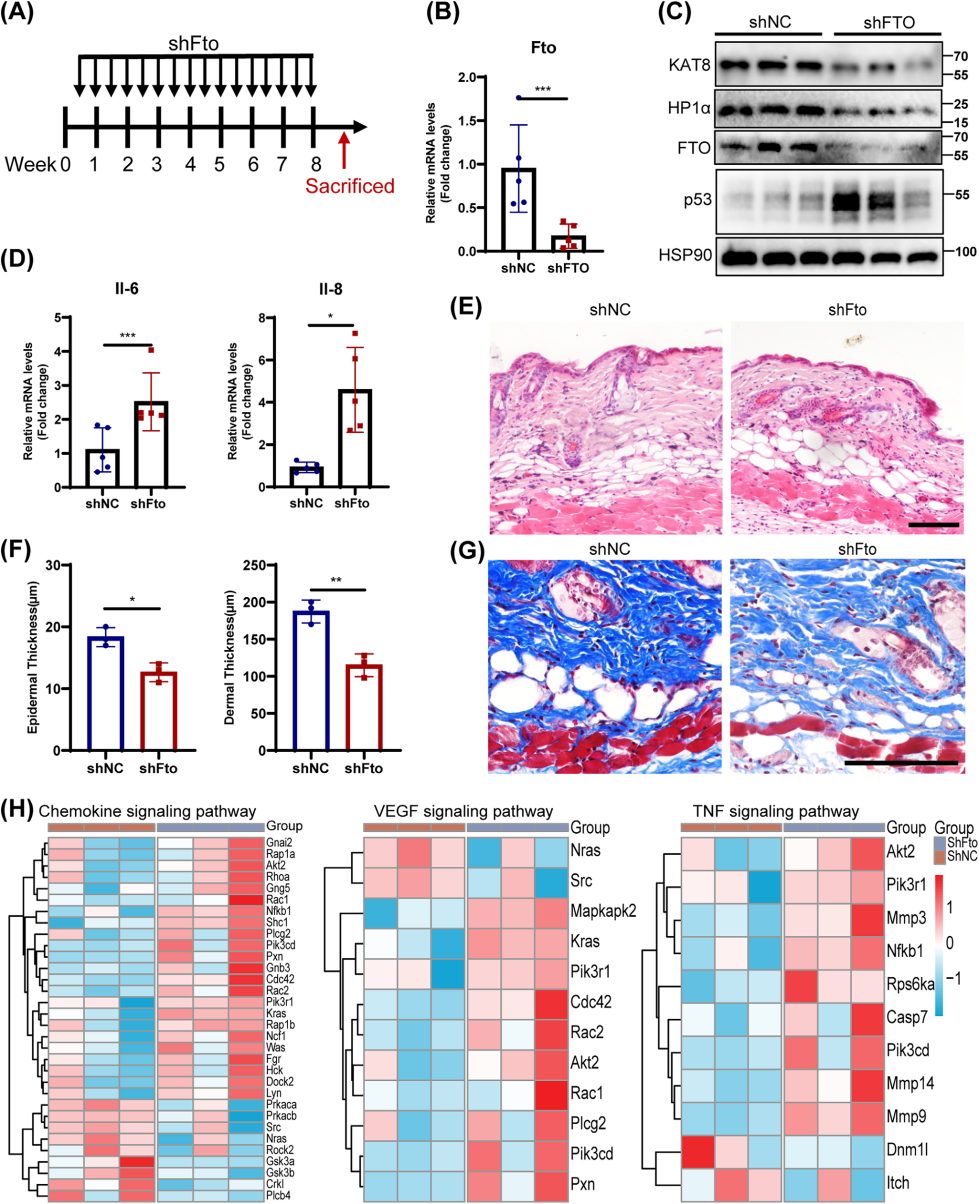
Fat mass and obesity‐associated protein (FTO)‐knockdown induced aging phenotype in mouse dorsal skin. (A) Schematic diagram of intradermal injection of shFTO‐lentivirus in C57BL/6 mice for 8 weeks. (B and D) The mRNA levels of Fto (B), Il‐6 and Il‐8 (D) in back skin tissues (*n* = 5 for each group). (C) The protein levels of FTO and p53. (E) Hematoxylin and eosin (H&E) staining of back skin tissues. (F) The skin thickness on the skin was assessed. (G) Masson staining of back skin tissues. (H) GSEA illustrated the enrichment of genes in Chemokine signaling pathway, VEGF signaling pathway and TNF signaling pathway in FTO‐depleted skin. Data are representative of at least three independent experiments. Scale bar, 100 µm. Data are shown as mean ± SEM. ns, not significant;*p < 0.05; **p < 0.01; ***p < 0.001; ****p < 0.0001.

The accumulation of senescent cells contributes to aging and age‐related pathological changes [33], thus we further evaluated the impact of FTO depletion on aging in vivo. Lentivirus expressing shRNA targeting Fto was intradermally injected into the back skin of wild‐type C57BL/6j mice to locally knockdown the expression of FTO in the skin (Figure 7A–C). The results showed that Fto deficiency induced aging phenotypes, including increased SASP (IL‐6, IL‐8) levels (Figure 7D) and p53 expression (Figure 7C), as well as decreased epidermal and dermal thickness and collagen fibers (Figure 7E,F). Furthermore, histological analysis revealed a significant increase in inflammatory cell infiltration in Fto‐deficient skin (Figure 7E). Moreover, the expression of KAT8 and HP1α were also reduced in Fto‐deficient skin tissue by western blot (Figure 7C). Notably, we also identified the level of H3K9me3 (another recognized heterochromatin index), and immunofluorescence staining showed decreased expression of H3K9me3 in the elderly as well as in Fto‐deficient skin fibroblasts of mice (Figure S10E,F).

Next, the proteomic analysis was performed on Fto‐depleted skin and the control skin. Compared with the control group, a total of 176 upregulated and 232 downregulated differentially expressed proteins (DEPs) were identified in Fto‐depleted skin tissues (adj. *p* < 0.05). GSEA‐GO analysis showed that biological processes such as “inflammatory response,” “immune response,” “tumor necrosis factor production,” “complement activation,” and “cytokine production” were significantly upregulated in Fto‐depleted skin compared with the control group (Figure S11A). In addition, GSEA‐KEGG analysis also showed several significantly upregulated pathways in Fto‐depleted skin compared with the control group including “IL‐17 signaling pathway,” “chemokine signaling pathway,” “cellular senescence,” “tumor necrosis factor signaling pathway,” and “VEGF signaling pathway” (Figure S11B). Among them, several core proteins (AKT2, CDC42, MMP3, et al.) of “Chemokine signaling pathway,” “VEGF signaling pathway,” and “TNF signaling pathway” were significantly altered (Figure 7H). Overall, Fto deficiency induced an aging microenvironment characterized by chronic inflammation and vascular disorders in vivo.

## 3 Discussion

As an important barrier between internal and external environment [34], skin aging is the most direct response to body aging [35, 36]. Many complex factors are involved in skin aging, among which epigenetic disorders make a difference [37, 38, 39]. Recent studies have highlighted the involvement of m6A modification in skin aging [40]. Here, we reveal that FTO (an m6A demethylase) exerts a protective effect against aging in skin. We identify KAT8 as a target of FTO in an m6A‐YTHDF2‐dependent manner. The ablation of KAT8 mediated by FTO decreases the acetylation of RSF1, which ultimately leads to loss of heterochromatin and onset of cellular senescence.

As previously mentioned, growing evidence indicated that epigenetic disorders were key contributors to skin aging. m6A modification, a prevalent epigenetic modification in the transcriptome, is the most common internal modification of mRNA [41]. The dynamic m6A levels are regulated by methyltransferase, demethylase and RNA‐binding proteins. Previous studies have reported that a variety of m6A modification regulators, such as ALKBH5 and METTL3, were involved in cellular senescence of multiple cell types [42, 43]. However, the relationship between m6A methylation and skin aging remains poorly understood. It has been shown that FTO, a demethylase, modulates the expression of downstream genes in an m6A‐dependent manner and is involved in multiple physiological processes. Studies have suggested that FTO knockdown or inhibition, through pharmacological inhibitors, disrupt the m6A modification of its target genes, ultimately impairing cell cycle progression [18, 44], suggesting a strong association between FTO and cellular senescence and even skin aging. In this study, we described the downregulation of FTO in aging skin tissues and HDFs, revealing its crucial role as a protective factor in HDF senescence and skin aging, mediated through m6A modification.

m6A regulatory proteins play diverse roles in various biological processes due to their specific key targets. Our study identified KAT8 as a new downstream target for FTO and further elucidated the important link between acetylation levels and the HDFs senescence. Acetylation, the process in which acetyl groups are transferred from acetyl‐CoA to the ε‐amino side chain of lysine residues by KATs, can be reversed by deacetylases (KDACs) [45]. Numerous studies have established a close relationship between acetylation levels and aging. For example, inhibition of acetyltransferase p300 or deacetyltransferase SIRT6 activity significantly induced mice aging or cell senescence [22, 46]. KAT8, also known as MOF, is a highly conserved histone acetyltransferase (HAT), which specifically recognizes lysine 16 of nucleosome histone H4 [47, 48]. Many studies have highlighted KAT8's crucial role in processes such as embryogenesis, cell proliferation, and DNA damage repair [49, 50, 51]. More recently, there has been growing interest in KAT8's involvement in aging. One study noted that overexpression of KAT8, or inhibition of H4K16 deacetylation, improved the aging‐related phenotype caused by Zmpste24 metalloproteinase gene deficiency in early mice [52]. Besides, the inhibitors of KAT6 (a family member with the same structure of KAT8) were found to induce cell cycle withdrawal and cell senescence [53]. Our experimental results supported the above studies, and we concluded that KAT8 was involved in FTO‐mediated skin aging by exerting its acetylase activity.

As an acetyltransferase, KAT8 has been certified to regulate the acetylation level of target proteins. Here, we confirmed that KAT8 enhanced the acetylation of lysine residues of RSF1, thereby delaying cell senescence. RSF1, a key component of the ISWI family of chromatin remodeling complexes, plays a critical role in DNA repair and chromatin remodeling [29, 30, 54]. Functional defects in chromatin remodeling complexes disrupt chromatin structure, leading to an overall loss and redistribution of heterochromatin, which often occurs in aging and cancer cells [31, 55]. Prior studies have noted a decrease in heterochromatin labeling levels (with HP1α as a key indicator) in older individuals or senescent cells [56, 57]. Intriguingly, senescence‐associated heterochromatic foci (SAHFs) are formed in specific regions of some senescent cells, which is not contradictory to the global loss of heterochromatin [58, 59]. In this work, we demonstrated that FTO/KAT8 deletion disrupted heterochromatin by downregulating acetylation level of RSF1, ultimately inducing skin aging and HDFs senescence.

This study also has certain limitations. First, while the acetylation proteomics focused on RSF1, it did not rule out the potential involvement of other target proteins, which may also play crucial roles in the process and warrant further investigation. Second, although our colocalization analysis and single‐cell analysis indicated that FTO is predominantly expressed in HDFs, we did not perform a fibroblast‐specific knockout of FTO in mice. This lack of a tissue‐specific knockout may limit our ability to fully understand the precise role of FTO in skin aging and HDFs senescence.

In summary, our work identified the epigenetic mechanism by which FTO regulates skin aging. FTO deficiency interfered with the stability of KAT8 mRNA, mediated by m6A‐dependent YTHDF2 recruitment, resulting in the reduced acetylation of RSF1 and the subsequent age‐related heterochromatin loss. Our study reveals the important role of FTO/KAT8/RSF1 axis in senescence, providing a potential way to improve skin aging.

## 4 Materials and Methods

### 4.1 Experimental Animals and Animal Treatment

Eight‐week‐old C57BL/6j (Slac Laboratory Animal Co., Shanghai, China) were used in this experiment. The mice were placed under specific pathogen‐free conditions with a 12‐h light/dark cycle at 24°C, with free access to food and water. All the mice were randomly divided into different groups, with no less than 5 mice in each group. All the experimental programs and procedures were approved by the Ethics Committee of Xiangya Hospital, Central South University, Hunan Province, China (IRB number 202103574).

For the model of systemic aging induced by D‐galactose (D‐gal) in mice, normal saline or high dose D‐gal (250 mg/kg/day; Selleck, China) was subcutaneously injected into the back skin of C57BL/6 with insulin syringe. All mice were injected daily for 8 weeks, and then the skin tissue was collected.

For in vivo gene knockdown in the dermis, 50 µL high titer lentivirus (greater than 5 × 10^8^ CFU/mL) (produced and collected as follows) was intradermically injected into the back skin of C57BL/6j mice with insulin syringes every 3 days for a total of 2 months, and then samples were collected for further experiments.

### 4.2 Bioinformatic Analysis

For the analysis of m6A‐related gene expression profiles of 16 widely accepted m6A RNA methylation regulators, a heatmap was generated to depict the expression of these regulators in the skin of healthy young individuals and individuals with premature senile syndrome. The “corrplot” package was used to assess the correlation between gene expression and age.

For single‐cell RNA sequencing data analysis, all datasets were sourced from public databases. The specific details of each dataset are outlined in Table S1. The Seurat package is used for data analytics. After quality control, data standardization, manifold approximation and projection (UMAP) dimensionality reduction, and clustering, groups of cells are identified and labeled according to different cell‐specific markers.

### 4.3 Skin Samples and Immunohistochemistry Analysis

All normal human skin samples were taken from volunteers' normal tissue after surgery, such as mole removal in the Department of Dermatology of our hospital, and was approved by the Ethics Committee of our hospital. Participants were divided into two groups according to their age: young (18–29 years old; *n* = 6; mean age 23.5), and old (70–82 years old; *n* = 6; mean age 76.5). For immunohistochemical determination, human skin slices were degreased, and rehydrated, and endogenous peroxidase was blocked. After sealing, the primary antibody was stained overnight at 4°C. After secondary antibody incubation, DAB coloration, and hematoxylin staining, the images were obtained after sealing. Anti‐FTO antibody (1:250, Abcam, catalog ab126605) was used.

### 4.4 Cell Culture and Treatments

The primary HDFs were isolated from the prepuce of voluntary and informed healthy donors aged 5–24 years old, from the Department of Urology, Xiangya Hospital, Central South University. HEK293T cells were purchased from ATCC. The cells were cultured in modified Eagle medium (DMEM; Gibco, USA) supplemented with 10% fetal bovine serum (Gibco, USA) and 1% penicillin–streptomycin, and incubated in 37°C and 5% CO_2_ incubator.

The primary HDFs were passaged continuously and stopped proliferating at about 50 population doubling level (PDL). We define young (Y, < 10 population doubling [PD]) and senile (O, > 40 PD) fibroblasts. To induce premature senility, young HDFs at about 70%–80% confluence were exposed to H_2_O_2_ (200 µM, 2 h) and UVA (10 J/cm^2^, 3 days), respectively.

Lentivirus infection produced stable knockout/overexpression cell lines. The sequences containing FTO shRNA or cDNA were cloned into PLKO.1 or PLVX vectors, respectively, and then the plasmid and other packaging plasmids were cotransfected into HEK293T cells. After 48 h and 72 h, the culture medium containing lentivirus particles was collected, a 0.45 µm filter was used to remove cell fragments, and then HDFs 24 h were infected. The infected cells were screened with 1 µg/mL puromycin, and the primers were listed in Table S2.

### 4.5 Total RNA Extraction, Real‐Time Quantitative PCR, and RNA‐seq Analysis

Total RNA was extracted from HDFs or skin tissues with TRIzol reagent (Thermo Fisher Scientific, USA). Following the manufacturer's instructions, cDNA was synthesized from 1000 ng of total RNA using the Reverse Transcription Kit (Takara, Japan) with gDNA digestion. qPCR was performed using the CFX Connect real‐time PCR system (Bio‐Rad, USA) and iTaq Universal SYBR Green Supermix (Vazyme, China). The sequences of qPCR primers are listed in Table S2.

For RNA‐seq of HDFs samples, total RNA was extracted from FTO‐knockdown HDF lines and shNC. Library preparation and transcriptome sequencing were carried out by OEBiotech Co., Ltd. (Shanghai, China). By the way, DEGs were identified with |log2FC| > 1 and adjusted *p* value < 0.05 in shFTO group, as compared to the shNC group.

### 4.6 Western Blotting

Cells or tissues were split on ice for 30 min in RIPA buffer (Beyotime Biotechnology, China), supplemented with a protease inhibitor cocktail (Cwbio, China). The extracted protein was denatured by heating in SDS loading buffer at 100°C, 5 min. Protein separation was performed using SDS‐polyacrylamide gel, followed by electroblotting onto a PVDF membrane. The membrane was then blocked with skim milk and incubated sequentially with the primary antibody, followed by a secondary antibody conjugated with HRP.

Anti‐HSP90 (1:5000; Proteintech, 60318‐1‐Ig), anti‐GAPDH (1:20,000; Bioworld, AP0066), anti‐FTO (1:10,000, Abcam, ab126605), anti‐Lamin B (1:5000; Proteintech, 66095‐1‐Ig), anti‐p53 (1:2,00; Santa Cruz, sc‐126), anti‐p21 (1:1000; Cell Signal Technology, 2947S), anti‐p16 (1:1000; Abcam, ab108349), anti‐KAT8 (1:1000, Abcam, ab200660), anti‐RSF1 (1:500, Abcam, ab109002), anti‐Flag (1:2000; Sigma, F3165), anti‐HA (1:1000; Cell Signal Technology, 3724S), and anti‐YTHDF2 (1:2000; Proteintech, 24744‐1‐AP). Secondary antibodies (anti‐rabbit, anti‐mouse) were purchased from Zybio Company.

### 4.7 RNA m6A Quantification Analysis and m6A Dot Blot Assay

The quality and concentration of RNA were detected by NanoDrop spectrophotometer. The m6A content was quantified using the EpiQuik m6A RNA Methylation Quantitative kit (colorimetry, Epyentek, USA). Total RNA was detected by RNA m6A dot blotting on a nylon membrane (GE Healthcare), then cross‐linked with ultraviolet (UV). After blocking, the m6A antibody (ab191606, Abcam, USA) was incubated overnight at 4°C, and m6A spots were analyzed by imaging system (Bio‐Rad, USA).

### 4.8 m6A RNA‐IP‐qRT–PCR (MeRIP‐qPCR)

The fragmented RNA was incubated with magnetic Dynabeads‐bound anti‐m6A antibody or normal IgG (negative control) (Abcam, USA) for m6A enrichment (Me‐RIP m6A Kit, Merck Millipore, USA). Subsequently, the expression was verified by qPCR. The primers are shown in Appendix Table 2.

### 4.9 Cell Proliferation Assays

The cells were seeded into a 96‐well plate and incubated in CCK‐8 (Cell Counting Kit‐8, C0038, Beyotime Biotechnology, China) diluted in culture medium at 37°C for 1 h. The absorbance was measured at 450 nm and continuously for 4 days.

### 4.10 Immunofluorescence

The cells (seeded on glass cover slides) or tissues (frozen sections) were fixed at room temperature with 4% paraformaldehyde for 10 min. Then, it was permeated for 15 min with 0.3% Triton X‐100. Following blocking with 5% donkey serum at room temperature (1 h), the cells or tissues were incubated with the primary antibody overnight at 4°C. Afterward, Alexa Fluor 488‐ or 594‐conjugated secondary antibodies (1:500, Thermo Fisher Scientific, USA) were applied at room temperature for 1 h. Nuclear DNA was stained using DAPI.

Anti‐ki67 (1:500, Abcam, catalog ab15580), anti‐FTO (1:250, Abcam, catalog ab126605), anti‐Vimentin (1:200, Abcam, catalog ab8978), 5‐ethynyl‐20‐deoxyuridine (EdU) (RiboBio, China, catalog C10310‐3), and anti‐H3K9me3 (1:500, Abcam, catalog ab176916) were used.

### 4.11 mRNA Stability Assay

Fibroblasts were harvested after treatment with actinomycin D (5 µg/mL) for 12, 6, 3, and 0 h. Total RNA was extracted according to the method previously described. The mRNA level of the target transcript was measured by qPCR. The mRNA half‐life was subsequently assessed by linear regression analysis.

### 4.12 Senescence‐Associated β‐Galactosidase Staining

After fixing in 10% formaldehyde for 10 min at room temperature and washing with PBS, the HDFs seeded in 6‐well plates were incubated overnight with β‐galactosidase staining solution (Beyotime Biotechnology, China) at 37°C. The images were captured by microscope and quantified by ImageJ software.

### 4.13 Hematoxylin–Eosin Staining and Masson Trichrome Staining

The skin tissue was fixed in 4% paraformaldehyde and embedded in paraffin. According to the standard procedure, paraffin sections (5 µm) were stained. The thickness of the epidermis, dermis was calculated by ImageJ software (NIH), collagen staining was evaluated.

### 4.14 Flow Cytometry

After different treatments, fibroblasts were digested by pancreatic enzymes (without EDTA), fixed and incubated with antibodies using the Annexin V Apoptosis Detection Kit (ThermoFisher, USA, BMS500FI‐300) and detected using flow cytometry (FACSCalibur, BD, San Jose, CA).

### 4.15 Dual‐Luciferase Reporter Assay

HEK293T cells were seeded in 24‐well plates. Each well was cotransfected with 400 ng shNC/shFTO, 100 ng of the firefly luciferase reporter plasmid pGL3 containing KAT8 3′UTR and 0.4 ng Renilla luciferase reporter using 3 uL of Lipofectamine 2000 (ThermoFisher, USA) for 48 h. Firefly and Renilla luciferase activities were measured using a dual‐luciferase reporter assay system.

### 4.16 m6A Methylation Site Prediction and Acetylation Binding Sites Prediction

The mRNA sequence was analyzed to predict the m6A recognition sites using SRAMP. The prediction results were based on three types of random forest models (KNN, binary, and spectral) to predict possible results.

### 4.17 Proteomic Profiling of Lysine Acetylation

HDFs from different treatments were dissolved in cell lysis buffers (8 M urea [Sigma, USA], protease inhibitor, TSA [Sigma, USA], and NAM [Sigma, USA]). The collected samples were trypsinized, and the peptides were freeze‐dried in a vacuum. Peptides were enriched with anti‐acetyllysine antibody conjugated with agarose beads (Santa Cruz, USA). The follow‐up quality control and operation process are carried out by Jingjie PTM BioLab (Hangzhou, China). The data obtained by LC–MS/MS analysis are searched for tandem mass spectra according to the human UniProt database.

### 4.18 Coimmunoprecipitation Assay

The cleavage buffer (Beyotime Biotechnology, China) supplemented with protease inhibitor (Cwbio, China) was used to cleave the cells. After preclearance with magnetic beads, the supernatant was incubated overnight with primary antibody or control IgG (Cell Signal Technology, USA). The magnetic beads with HA or Flag tags were directly combined with protein overnight, and then precipitated by the magnetic frame were denatured and determined downstream by immunoblotting.

### 4.19 LC–MS/MS Analysis and Proteomics Analysis

For protein samples were extracted from skin tissue, and 30 µg of each sample were processed following the instructions of proteome pretreatment preparation kit (Omicsolution, China). Subsequently, peptides were isolated and analyzed by the Vanquish Neo HPLC system (Thermo, USA) and the Orbitrap Exploris 480 mass spectrometer (Thermo, USA), respectively. Next, original DIA data source was analyzed with Spectronaut (v18).

Differential expression analysis was performed on control group and shFto group. Proteins with *p* values < 0.05 and |log2 (FoldChange)| ≥ 1 were adjusted to differentially expressed proteins. And GSEA enrichment analysis was performed using ClusterProfiler R package.

### 4.20 Flow Cytometry Analysis of Apoptosis

The cells were processed according to the operating instructions of the Annexin‐V fluorescein isothiocyanate/propidium iodide (PI) kit (4A BIOTECH, China). Subsequently, flow cytometry was performed by flow cytometer (FACSCalibur, BD, San Jose, CA).

### 4.21 Statistical Analysis

GraphPadPrism (version 8.0.0) was used for statistical analysis. All the data were expressed as the mean of at least three independent experiments. Unpaired student *t*‐test was employed for comparison between two groups, while one‐way analysis of variance (ANOVA) was used for multigroup comparison.

## Author Contributions

Fan Wang: Conceptualization, Formal analysis, Data curation, Validation, Methodology, Investigation, Writing—original draft. Lei Zhou: Conceptualization, Data curation, Methodology, Investigation, Formal analysis, Validation. Yun Zhong: Investigation, Methodology. Yisheng Cai: Methodology, Software, Visualization. Xin Meng: Investigation, Methodology. Mengting Chen: Methodology. Rui Mao: Software, Visualization. Xin Xiao: Methodology, Software, Visualization. Caitan Yi: Methodology. Yi Guo: Methodology. Hongfu Xie: Resources, Supervision. Yiya Zhang: Conceptualization, Formal analysis, Project administration, Supervision, Writing – review & editing. Ji Li: Funding acquisition, Resources, Project administration, Supervision. All the authors read and approved the final manuscript.

## Ethics Statement

Both human and animal studies were approved by the Ethics Committee of Xiangya Hospital, Central South University, Hunan Province, China (approval number: 201708973; 202103574).

## Conflicts of Interest

The authors declare no conflicts of interest.

## Supporting information



Supporting Information

## Data Availability

The RNA‐seq transcriptome data of fibroblasts of healthy young individuals and premature senile syndrome individuals were obtained from GSE113957 dataset (https://www.ncbi.nlm.nih.gov/geo/), and the MeRIP‐seq data of FTO were obtained from GSE124509 dataset. The transcriptome data (RNA‐seq data of FTO‐depleted human dermal fibroblasts) are openly available in the NCBI database (accession no. PRJNA1199897), acetylated proteome data of human dermal fibroblasts and proteome data of mice skin tissues are publicly available in the Proteome Xchange‐iProX database (accession nos. PXD059135 and PXD059134, respectively).
