# Correction to “Depletion of Fat Mass and Obesity‐Associated Protein (FTO) Drives Heterochromatin Loss via Lysine Acetyltransferase 8 (KAT8)‐Mediated Remodeling and Spacing Factor 1 (RSF1) Acetylation in Skin Aging”

**DOI:** 10.1002/mco2.70384

**Published:** 2025-09-18

F. Wang, L. Zhou, Y. Zhong, et al., “Depletion of Fat Mass and Obesity‐Associated Protein (FTO) Drives Heterochromatin Loss via Lysine Acetyltransferase 8 (KAT8)‐Mediated Remodeling and Spacing Factor 1 (RSF1) Acetylation in Skin Aging,” *MedComm* 6, no. 7 (2025): e70205, https://doi.org/10.1002/mco2.70205.

In the process of checking the raw data [1], the authors noticed an inadvertent mistake occurring in Figure 4C that needed to be corrected after the online publication of the article. The correct result should be as shown below. The authors apologize for these oversights and declare that this correction does not affect the description, interpretation, or conclusions detailed in the original manuscript.

**FIGURE 4:**
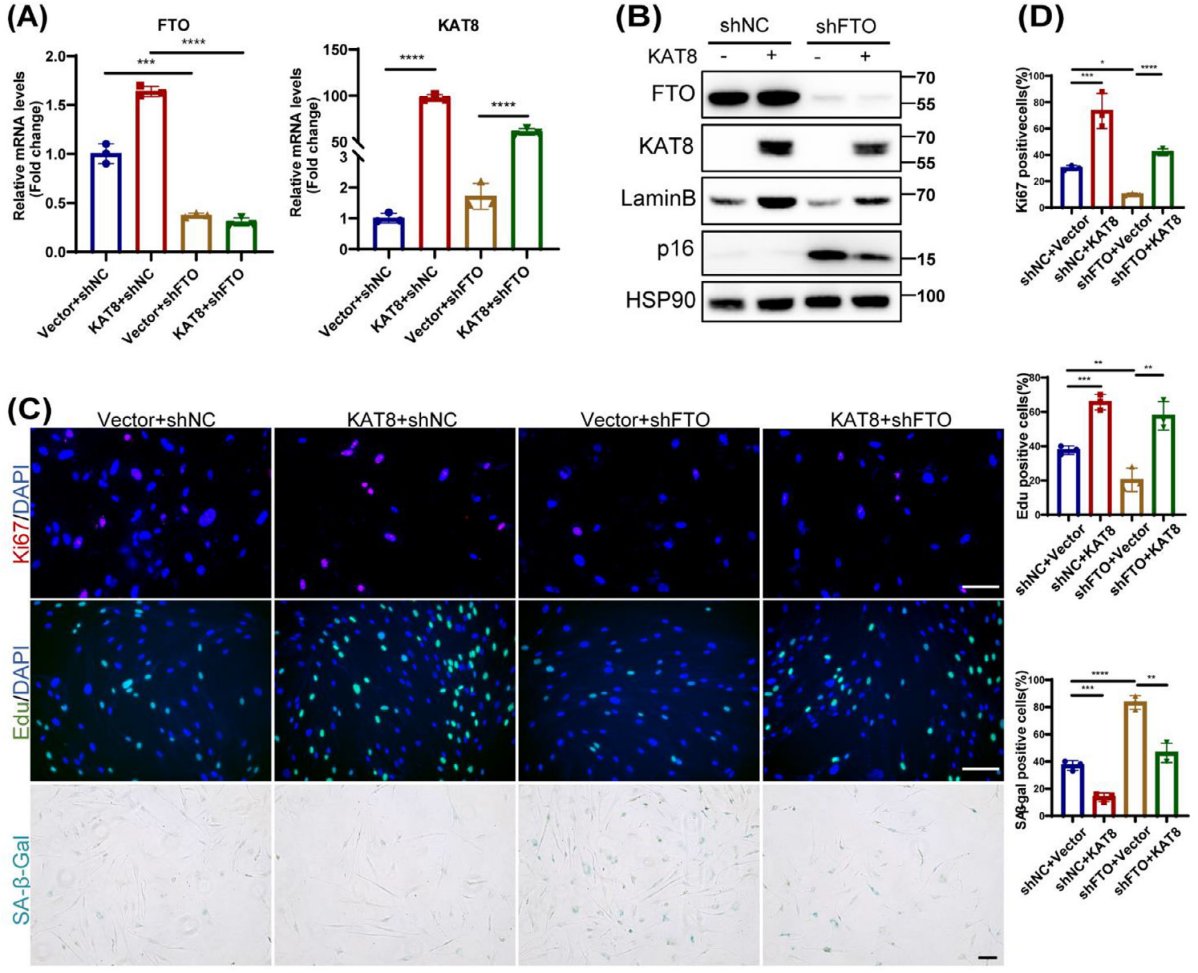
KAT8 was involved in FTO‐mediated cellular senescence in HDFs. (A) The mRNA level of FTO and KAT8 in FTO‐deprived HDFs following KAT8‐lentivirus transfection. (B) Expression of p53, p21, and p16 were verified in protein level. (C) Immunofluorescence staining of Ki67, EdU, and SA‐β‐Gal staining were conducted. (D) represents the statistical results of (C). Data are representative of at least three independent experiments. Scale bar, 100 µm. Data are shown as mean ± SEM. ns, not significant;*p < 0.05; **p < 0.01; ***p < 0.001; ****p < 0.0001.

1. F. Wang, L. Zhou, Y. Zhong, et al. “Depletion of Fat Mass and Obesity‐Associated Protein (FTO) Drives Heterochromatin Loss via Lysine Acetyltransferase 8 (KAT8)‐Mediated Remodeling and Spacing Factor 1 (RSF1) Acetylation in Skin Aging,” *MedComm* 6, no. 7 (2025): e70205. https://doi.org/10.1002/mco2.70205.

