# Supplementary material for: Depletion of Fat Mass and Obesity‐Associated Protein (FTO) Drives Heterochromatin Loss via Lysine Acetyltransferase 8 (KAT8)‐Mediated Remodeling and Spacing Factor 1 (RSF1) Acetylation in Skin Aging
Source: MedComm (2020). 2025 Jul 9;6(7):e70205. doi: 10.1002/mco2.70205 (PMC12238725; doi:10.1002/mco2.70205)
Supplement: Supplementary file 1 — Supporting Information [file MCO2-6-e70205-s001.docx]

**Depletion of Fat Mass and Obesity-Associated Protein (FTO) drives heterochromatin loss via Lysine Acetyltransferase 8 (KAT8)-mediated Remodeling and Spacing Factor 1 (RSF1) acetylation in skin aging**

Fan Wang^1,2,3#^, Lei Zhou^2,4#^, Yun Zhong^2,3,5^, Yisheng Cai^1,2,3^, Xin Meng^1,2,3^, Mengting Chen^1,2,3^, Rui Mao^1,2,3^, Xin Xiao^1,2,3^, Caitan Yi^1,2,3^, Yi Guo^1,2,3^, Hongfu Xie^1,2,3^, Yiya Zhang^1,2,3*^, and Ji Li^1,2,3*^

**Affiliations:**

1. Department of Dermatology, Xiangya Hospital, Central South University, Changsha, China.

2. Hunan Key Laboratory of Aging Biology, Xiangya Hospital, Central South University, Changsha China.

3. National Clinical Research Center for Geriatric Disorders, Xiangya Hospital, Central South University, Changsha, China.

4. Department of Dermatology, the Third Affiliated Hospital, Sun Yat-sen University, Guangzhou, P.R. China.

5. Department of Dermatology, Nanfang Hospital, Southern Medical University, Guangzhou, China.

^#^ These authors contributed equally to this work：Fan Wang and Lei Zhou

***Correspondence**: Ji Li and Yiya Zhang, Department of Dermatology, Xiangya Hospital, Central South University, Changsha, China.

Email: [liji_xy@csu.edu.cn](mailto:liji_xy@csu.edu.cn); yiya0108@csu.edu.cn.

**Supplementary Materials**

Table S1. details of each dataset.

Table S2. List of primer sequences in this study.

Table S3. acetylated differential proteins of acetylated proteome.

Figure S1. Expression of m6A RNA methylation regulators in fibroblasts of healthy young individuals and individuals with premature senility syndrome.

Figure S2**.** Single-cell RNA Sequencing of FTO in human skin tissues.

Figure S3**.** FTO co-localized with dermal fibroblasts in young skin.

Figure S4**.** FTO deficiency or inhibition had no effect on apoptosis of HDFs.

Figure S5**.** RNA-seq results revealed the Differentially expressed genes (DEGs) between FTO-deficient HDFs and control group.

Figure S6**.** The m6A sites of KAT8 mRNA 3'UTR were predicted in a motif-dependent m6A site predictor SRAMP (<http://www.cuilab.cn/sramp>)

Figure S7**.** Expression of KAT8 regulated senescent phenotype of HDFs.

Figure S8**.** FTO/KAT8 regulated the levels of SASP.

Figure S9**.** Knockdown of RSF1 induced cellular senescence in young HDFs.

Figure S10**.** Expression of heterochromatin in HDFs or skin tissues after different treatments.

Figure S11**.** Proteomic Data Analysis of FTO-depleted skin tissues.

## Table S1. details of each dataset.

|  | Subject ID | Sex | Age at Biopsy |
| --- | --- | --- | --- |
| GSE138669 | SC1nor | Male | 63 |
|  | SC4nor | Male | 54 |
|  | SC18nor | Female | 66 |
|  | SC32nor | Female | 23 |
|  | SC33nor | Female | 62 |
|  | SC34nor | Male | 24 |
|  | SC50nor | Male | 64 |
|  | SC68nor | Female | 48 |
|  | SC124nor | Male | 54 |
|  | SC125nor | Male | 61 |
| GSE156326 | skin1 | Female | 30 |
|  | skin2 | Female | 36 |
|  | skin3 | Female | 43 |
| GSE162183 | Ctrl1 | Female | 32 |
|  | Ctrl2 | Male | 23 |
|  | Ctrl3 | Male | 47 |
| GSE130973 | y1 | na | 25 |
|  | y2 | na | 27 |
|  | o1 | na | 53 |
|  | o2 | na | 70 |
|  | o3 | na | 69 |
| aging_2018 | SC1control | male | 63 |
|  | SC14control | male | 54 |
|  | SC18control | female | 66 |
|  | SC32control | female | 23 |
|  | SC33control | female | 62 |
|  | SC34control | male | 24 |

## Table S2. List of primer sequences in this study.

| Gene symbol | Forward primer | Reverse primer |
| --- | --- | --- |
| **Primers for qPCR:** | | |
| Human GAPDH | GGAGCGAGATCCCTCCAAAAT | GGCTGTTGTCATACTTCTCATGG |
| Human FTO | AACACCAGGCTCTTTACGGTC | TGTCCGTTGTAGGATGAACCC |
| Human KAT8 | GTCACGGTGGAGATCGGAGA | CCCTCCTGGTCGTTCACTC |
| Human RSF1 | GGATGCCGATACTATGCGTCT | GCCAACTCGTTTCGATTTCTGA |
| Human IL-1β | ATGATGGCTTATTACAGTGGCAA | GTCGGAGATTCGTAGCTGGA |
| Human IL-6 | ACTCACCTCTTCAGAACGAATTG | CCATCTTTGGAAGGTTCAGGTTG |
| Human IL-8 | TTTTGCCAAGGAGTGCTAAAGA | AACCCTCTGCACCCAGTTTTC |
| Human MMP3 | AGTCTTCCAATCCTACTGTTGCT | TCCCCGTCACCTCCAATCC |
| Mouse GAPDH | AGGTCGGTGTGAACGGATTTG | GGGGTCGTTGATGGCAACA |
| Mouse FTO | AAGCATGGCTGCTTGTTTCG | CTGGTGTCTCGATGTCCCAA |
| **Primer for shRNA:** | | |
| Human shNC  Mouse shNC | CCGGCAACAAGATGAAGAGCACCAACTCGAGTTGGTGCTCTTCATCTTGTTGTTTTTG | AATTCAAAAACAACAAGATGAAGAGCACCAACTCGAGTTGGTGCTCTTCATCTTGTTG |
| Human shFTO#1 | CCGGTCACGAATTGCCCGAACATTACTCGAGTAATGTTCGGGCAATTCGTGATTTTTG | AATTCAAAAATCACGAATTGCCCGAACATTACTCGAGTAATGTTCGGGCAATTCGTGA |
| Human shFTO#2 | CCGGTCACCAAGGAGACTGCTATTTCTCGAGAAATAGCAGTCTCCTTGGTGATTTTTG | AATTCAAAAATCACCAAGGAGACTGCTATTTCTCGAGAAATAGCAGTCTCCTTGGTGA |
| Human shKAT8#1 | CCGGCGAAATTGATGCCTGGTATTTCTCGAGAAATACCAGGCATCAATTTCGTTTTTG | AATTCAAAAACGAAATTGATGCCTGGTATTTCTCGAGAAATACCAGGCATCAATTTCG |
| Human shKAT8#2 | CCGGGCAAGATCACTCGCAACCAAACTCGAGTTTGGTTGCGAGTGATCTTGCTTTTTG | AATTCAAAAAGCAAGATCACTCGCAACCAAACTCGAGTTTGGTTGCGAGTGATCTTGC |
| Human shYTHDF2 | CCGGGATGGATTAAACGATGATGATCTCGAGATCATCATCGTTTAATCCATCTTTTTG | AATTCAAAAAGATGGATTAA  ACGATGATGATCTCGAGATCATCATCGTTTAATCCATC |
| Human shKAT8（3'UTR） | CCGGGGCTCCCAGCCTGTAAATATGCTCGAGCATATTTACAGGCTGGGAGCCTTTTTG | AATTCAAAAAGGCTCCCAGCCTGTAAATATGCTCGAGCATATTTACAGGCTGGGAGCC |
| Human shRSF1#1  （3'UTR） | CCGGCCAGTTCTGAACTTTGAAGATCTCGAGATCTTCAAAGTTCAGAACTGGTTTTTG | AATTCAAAAACCAGTTCTGAACTTTGAAGATCTCGAGATCTTCAAAGTTCAGAACTGG |
| Human shRSF1#2  （3'UTR） | CCGGGTGCTAATTTATTCCACGGTACTCGAGTACCGTGGAATAAATTAGCACTTTTTG | AATTCAAAAAGTGCTAATTTATTCCACGGTACTCGAGTACCGTGGAATAAATTAGCAC |
| Mouse shFTO | CCGGGATGATGAAGTGGACCTTAAGCTCGAGCTTAAGGTCCACTTCATCATCTTTTTG | AATTCAAAAAGATGATGAAGTGGACCTTAAGCTCGAGCTTAAGGTCCACTTCATCATC |
| **Primer for plasmids:** | | |
| FTO | aagctgggcggtggtggatccATGAAGCGCACCCCGACT | tgcaccggtactagttctagaCTAGGGTTTTGCTTCCAGAAGC |
| KAT8 | ggcggtggtggatccgaattcATGGCGGCACAGGGAGCT | tgcaccggtactagttctagaTCACTTCTTGGAGAGCTTGACTTG |
| RSF1 | ggatctatttccggtgaattcATGGCGACGGCGGCGGCA | tgggtacgcggccgctctagaTAACTGTTCACTGTTACAGACATAATCAAC |
| **Primers for KAT8 functional site-directed mutagenesis:** | | |
| KAT8-K274R | GGACCATAgGACACTGTACTTTGACGTGGAGCC | ACAGTGTCcTATGGTCCAGGAAAAGCTTGGCC |
| **Primers for site-directed mutagenesis of KAT8 m6A binding site:** | | |
| KAT8 mRNA 3'UTR | ctggcctaactggccggtaccTCCCAAACCAGTCAGACCAGC | ccagatcttgatatcctcgagTCTTGGCCTCCCCTTCCC |
| KAT8 m6A binding site 1 mutation: | CACAGTGGcCTCCGTCTGCCTCAAGTGGGCAC | AGACGGAGgCCACTGTGGGGAGAAGGGGCGGG |
| KAT8 m6A binding site 2 mutation: | / | ccagatcttgatatcctcgagTCTTGGCCTCCCCTTCCCCTCCAAAGgCCA |
| **Primers for site-directed mutagenesis of RSF1 acetylation site:** | | |
| RSF1-K1050R | CCGAGGAAgAGATATCTCCACCATCACAGGTCA | AGATATCTcTTCCTCGGCCAACTCCTCCTCCA |
| RSF1-K1061R | TCGTGGGAgAGACATCTCTACTATTTTGGATGAAGAAA | AGATGTCTcTCCCACGATGACCTGTGATGGTG |
| RSF1-2KR | tccaccatcacaggtcatcgtgggagAGACATCTCTACTATTTTGGATGAAGAAA | atgacctgtgatggtggagatatctcTTCCTCGGCCAACTCCTCC |

## Table S3 acetylated differential proteins of acetylated proteome.

| Names | total | elements |
| --- | --- | --- |
| KAT8vsOld  OldvsYoung  shFTOvsYoung | 21 | SLC25A6、H2BC5、H2BC4、TBC1D24、H2BU1、GALR1、HNRNPA1、CORO1C、H2BC18、H2BC13、MPHOSPH10、RSF1、EP300、JPT2、NPM1、INTS4、H2BC9、NUP214、SOD1、RRS1、MYO18A |
| KAT8vsOld OldvsYoung | 27 | H2AC14、PPP1CC、ALDOA、RPL12、HSP90B1、ANAPC7、H2AZ2、TKT、USP7、H3-3A、HYPK、VRK2、PTBP1、PSMD7、TRIM33、HNRNPK、DCPS、TCP1、PCNT、HNRNPC、HSP90AA1、RPN1、PKM、SLC25A5、ATP5F1A、FASN、JADE1 |
| OldvsYoung shFTOvsYoung | 8 | GOT2、SDCBP、HADH、PAPOLA、ICAM1、PDS5A、HSPA1B、ACTG1 |
| KAT8vsOld shFTOvsYoung | 2 | H4C1、MKI67 |
| OldvsYoung | 13 | HSP90AB1、ATAD1、NME2、ETFA、COPA、CKB、CLTC、RAN、RRNAD1、H2AZ1、RPS4X、KAT6A、TRO |
| KAT8vsOld | 8 | RBBP4、DNAJA1、EEF2、EIF5A、PCBP1、SAFB、USF3、UBA1 |
| shFTOvsYoung | 25 | SESTD1、WRNIP1、PHF5A、NCL、HSPB1、BAZ1B、VDAC1、MED6、PDIA3、MYL6、GAPDH、NUDC、ARHGAP29、PFN1、KMT2A、CCT8、SPTBN1、SRP54、HSPA9、PSMC2、RBBP7、P4HB、ATP1B3、NONO、RPS24 |


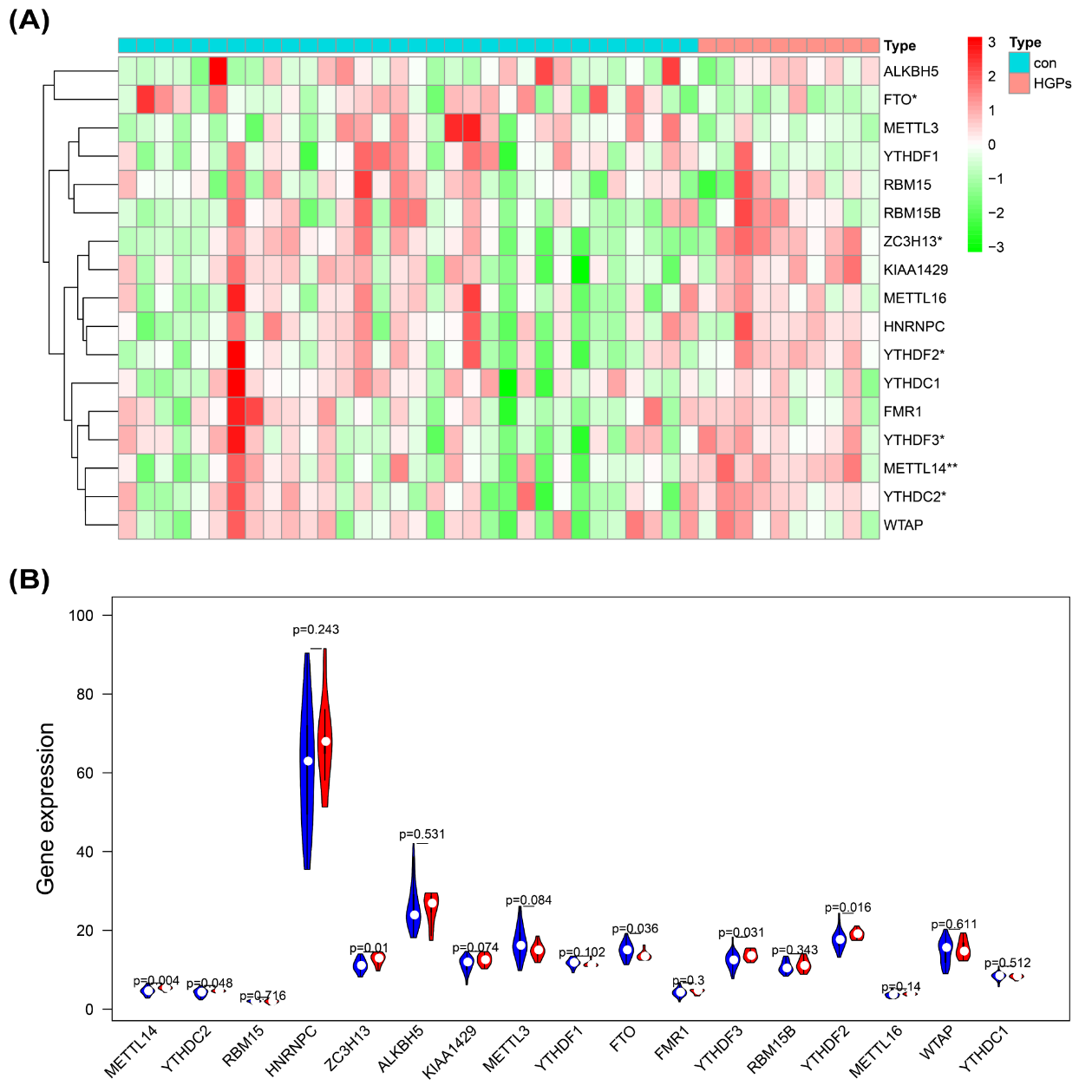


**Figure S1.**

Expression of m6A RNA methylation regulators in fibroblasts of healthy young individuals and individuals with premature senility syndrome.

(A), Heatmaps of expression levels of the m6A RNA methylation regulators from GSE113957. (B), Violin diagrams corresponding to (A).


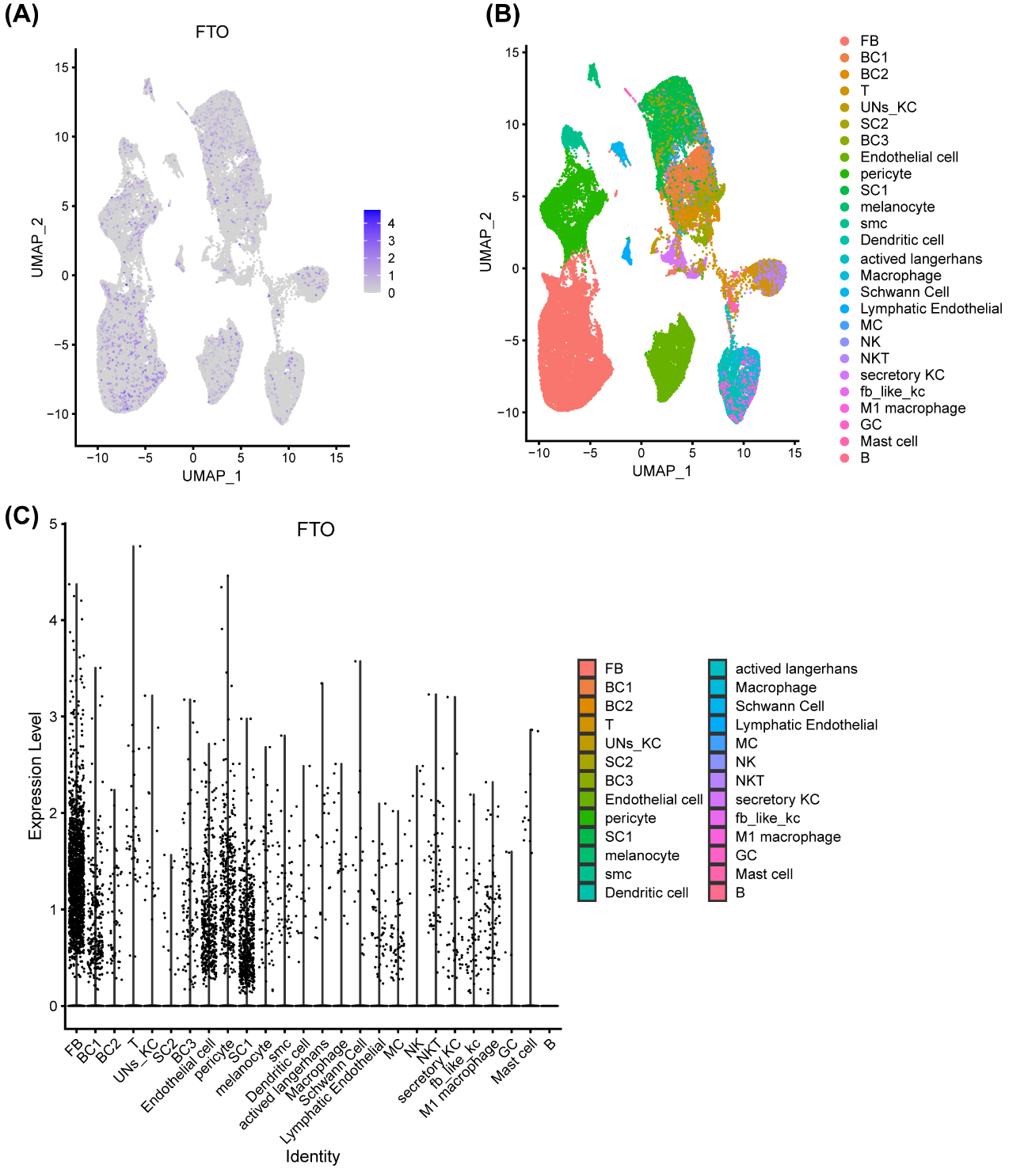


**Figure S2.**

Single-cell RNA Sequencing of FTO in human skin tissues.

(A), Diplot of single cell clustering. (B), Featureplot of FTO. (C), Vlnplot of FTO in each cell.

FB, Fibroblasts; BC, Basal Cells; B, B Cells; T, T Cells; KC, Keratinocytes; UNs_KC, Undefined Keratinocytes; SMC, Smooth Muscle Cells; MC, Mast Cells; NK, Natural Killer Cells; NKT, Natural Killer T Cells; GC, Germinal Center cells.


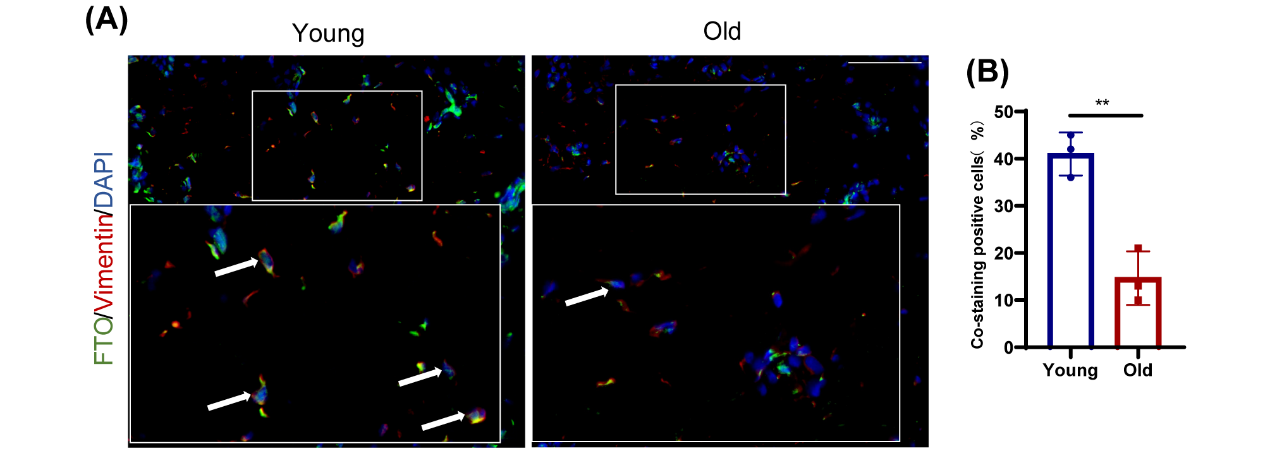


**Figure S3.**

FTO co-localized with dermal fibroblasts in young skin.

(A), Immunofluorescence co-staining of FTO and Vimentin in skin tissues of young (n = 6, mean age 23.5) and old individuals (n = 6, mean age 76.5). (B), the co-staining density was calculated. White arrows indicate FTO staining positive fibroblasts. Data are representative of at least three independent experiments. Scale bar, 100 μm. Data are shown as mean ± SEM. *P < 0.05; **P < 0.01; ***P < 0.001; ****P < 0.0001; ns, not significant.


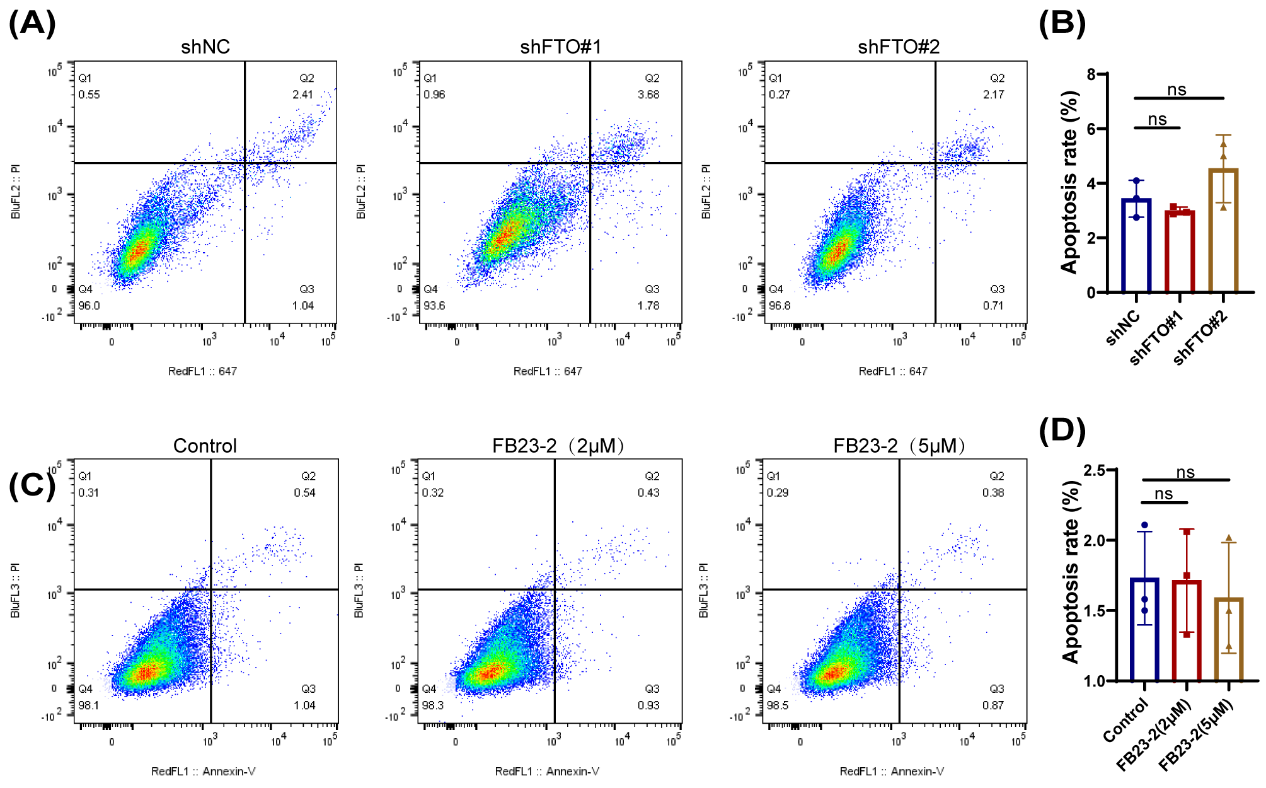


**Figure S4.**

FTO deficiency or inhibition had no effect on apoptosis of HDFs.

(A and C), Apoptosis was detected by flow cytometry in FTO-depleted HDFs (A) or HDFs treated with FB23-2 (C). (B and D), Statistical analysis of (A) and (C), respectively. Data are representative of at least three independent experiments. Data are shown as mean ± SEM. ns, not significant.


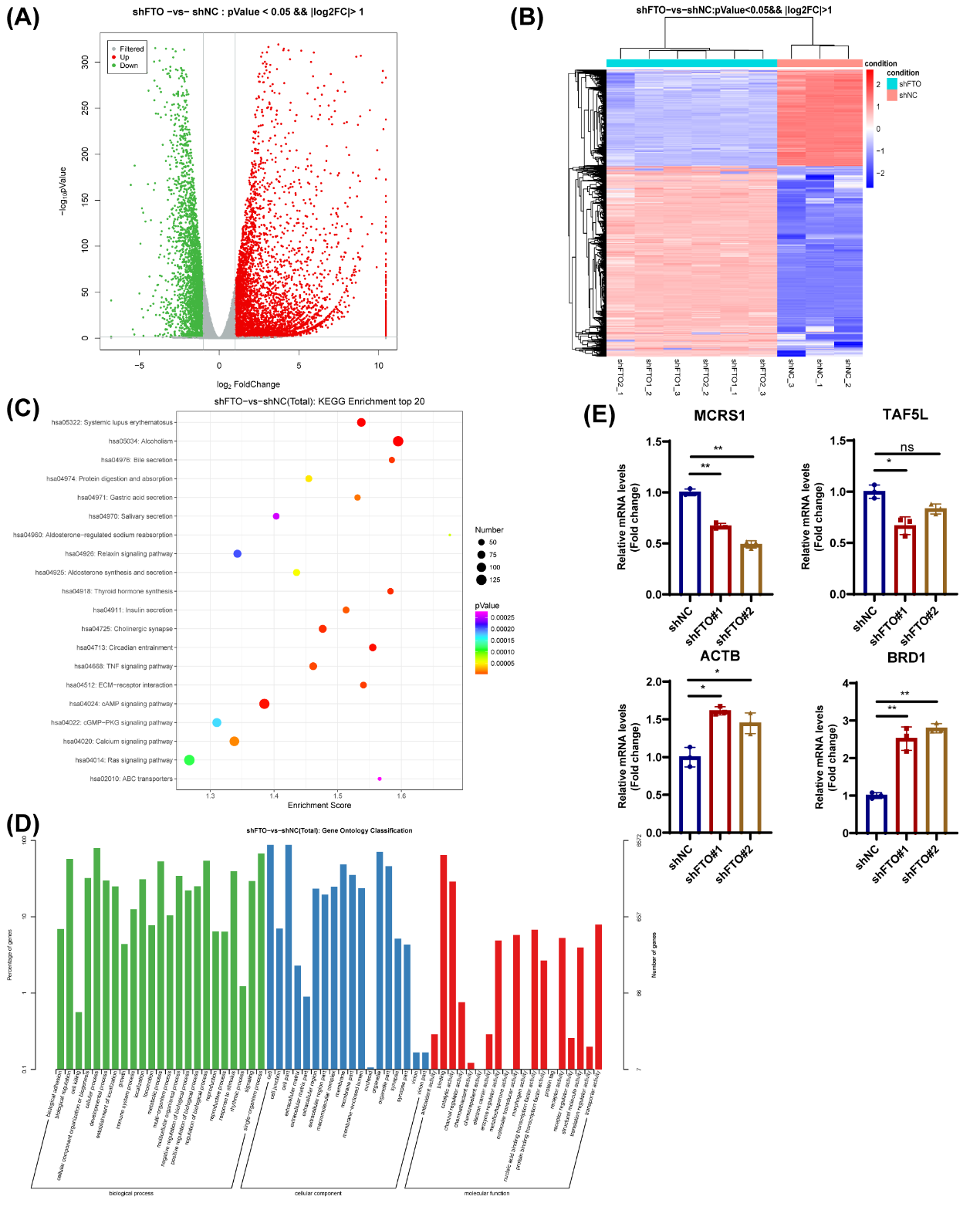


**Figure S5.**

RNA-seq results revealed the Differentially expressed genes (DEGs) between FTO-deficient HDFs and control group.

(A and B), The volcano map and heatmap of DEGs; Red or green in the diagram shown high or low expression, respectively. (C), GO analysis indicated the biological processes (BP), cellular components (CC), and molecular functions (MF) of DEGs. (D), KEGG pathway and the network of enrichment pathway of DEGs. (E), Expression of MCRS1, TAF5L, ACTB and BRD1 was examined by qPCR in HDFs following the knockdown of FTO. Data are representative of at least three independent experiments. Data are shown as mean ± SEM. *P < 0.05; **P < 0.01; ***P < 0.001; ****P < 0.0001; ns, not significant.


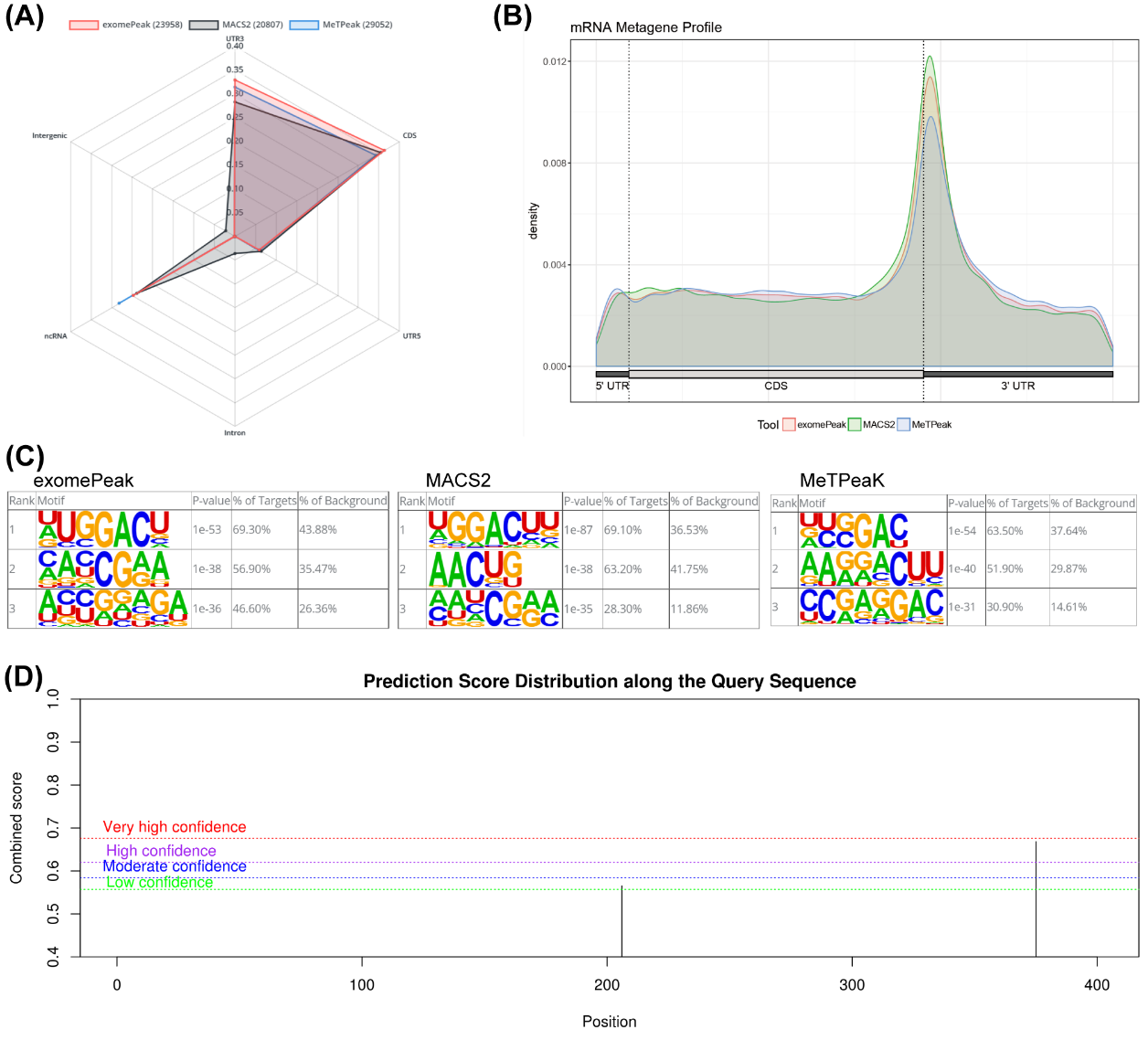


**Figure S6.**

The m6A sites of KAT8 mRNA 3'UTR were predicted in a motif-dependent m6A site predictor SRAMP (<http://www.cuilab.cn/sramp>)

(A, B and C), m6A-seq shows the characteristics of m6A peak. The Ray Datuk distribution of Peak on mRNA results (A), the Metagene Profile diagram of distribution of Peak on mRNA structure (B), and the results of motif analysis (C). (D), SRAMP predicts m6A modification sites in KAT8 mRNA 3'UTR sequences.


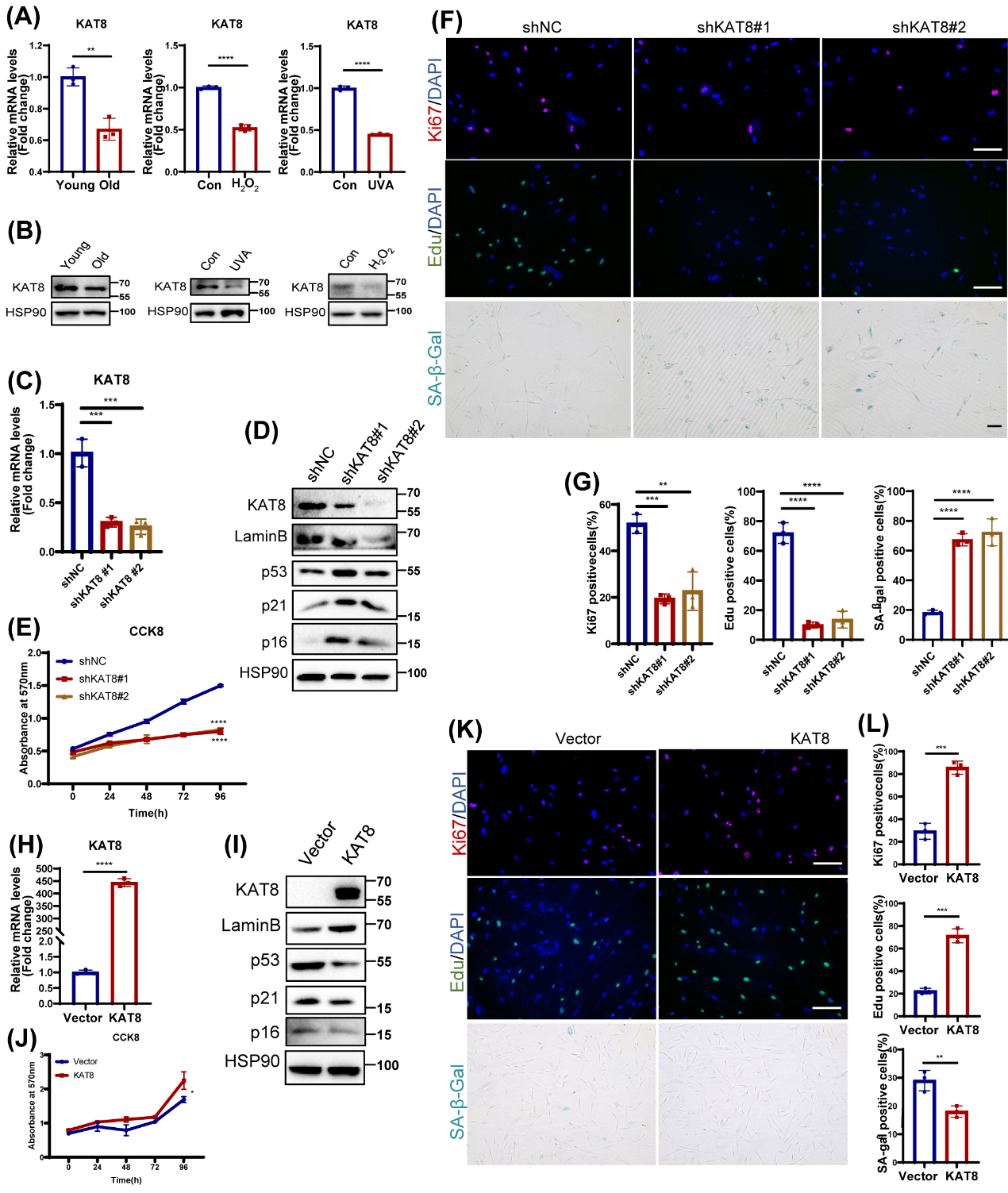


**Figure S7.**

Expression of KAT8 regulated senescent phenotype of HDFs.

(A and B), qPCR assay (A) and western blot assay (B) revealed the KAT8 expression in different senescent HDFs models. Young HDFs (PD < 10) were infected with shKAT8 or negative control shNC lentivirus, and senescent HDFs (PD > 40) were transfected with FTO or negative control lentivirus. (C and H), The mRNA expression of KAT8 following knockdown or overexpression of KAT8. (D and I), The protein levels of KAT8, Lamin B, p53, p21, and p16. CCK8 assay (E and J), immunofluorescence staining, and SA-β-Gal staining (F and K) were conducted. (G and L), Positive staining cells were quantified. Data are representative of at least three independent experiments. Scale bar, 100 μm. Data are shown as mean ± SEM. *P < 0.05; **P < 0.01; ***P < 0.001; ****P < 0.0001; ns, not significant.


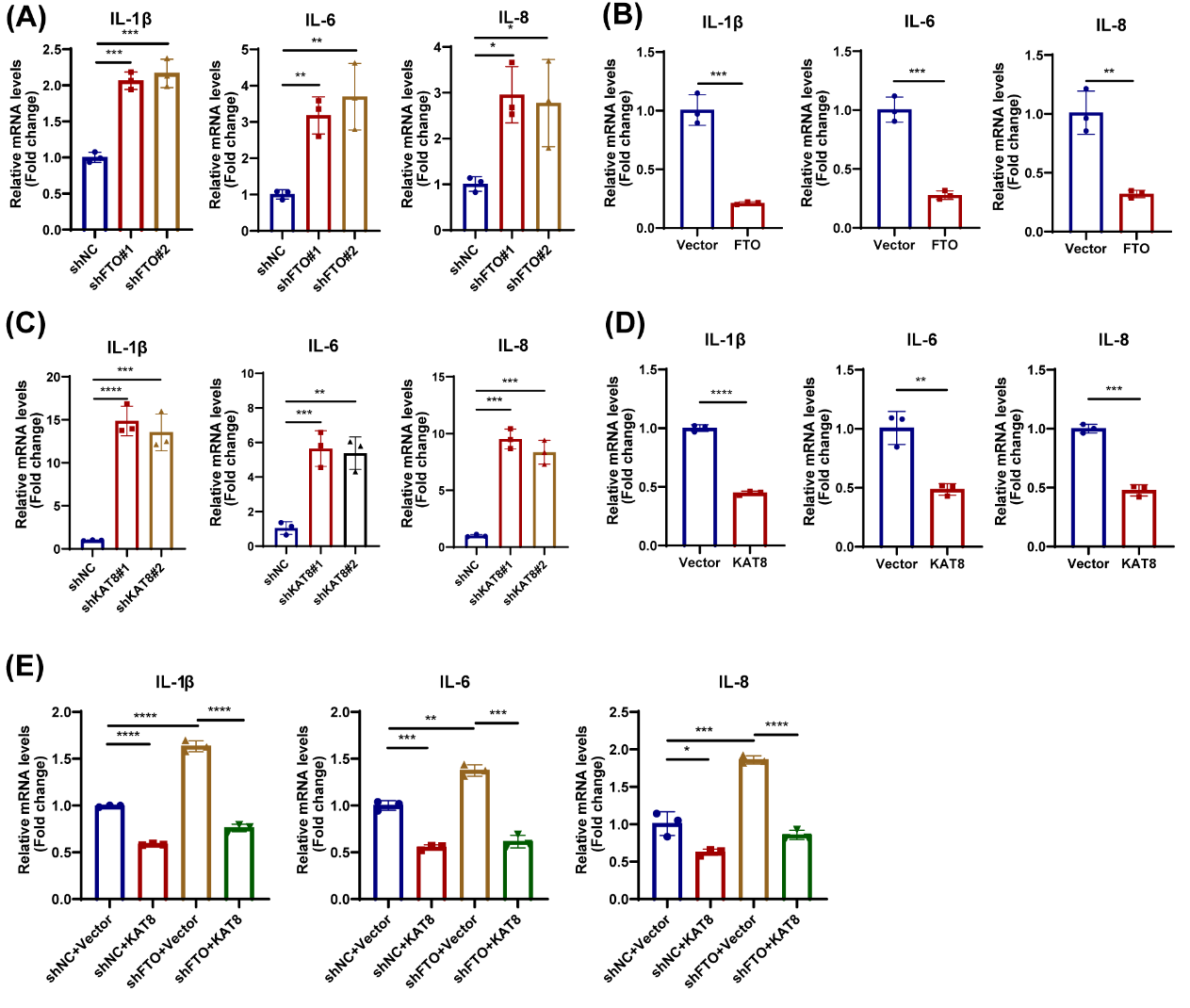


**Figure S8.**

FTO/KAT8 regulated the levels of SASP.

The mRNA levels of IL-1β, IL-6 and IL-8 by qPCR in HDFs upon knockdown of FTO (A) or KAT8 (C), ectopic of FTO (B) or KAT8 (D), and rescue assay (E). Data are representative of at least three independent experiments. Data are shown as mean ± SEM. *P < 0.05; **P < 0.01; ***P < 0.001; ****P < 0.0001; ns, not significant.


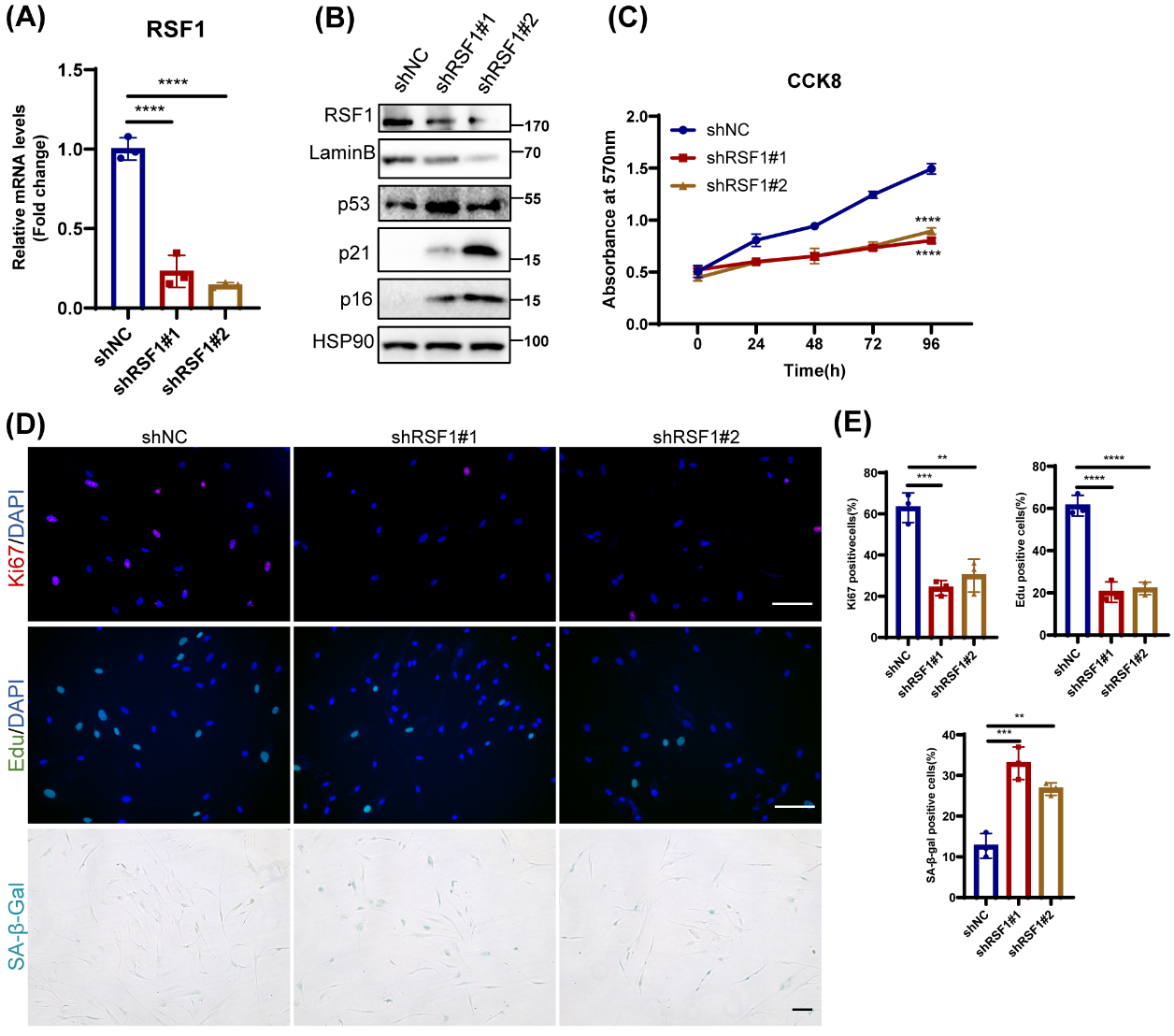


**Figure S9.**

Knockdown of RSF1 induced cellular senescence in young HDFs.

mRNA (A) and protein (B) level of RSF1 in HDFs after shRSF1-lentivirus transfection. (B), The expression of senenscent index (Lamin B, p53, p21, and p16). (C), Cell proliferation measured by CCK8. (D), Immunofluorescence staining of Ki67, Edu, and SA-β-Gal staining in HDFs upon knockdown of RSF1, and the percentage of positive staining cells were quantified (E). Data are representative of at least three independent experiments. Scale bar, 100 μm. Data are shown as mean ± SEM. *P < 0.05; **P < 0.01; ***P < 0.001; ****P < 0.0001; ns, not significant.


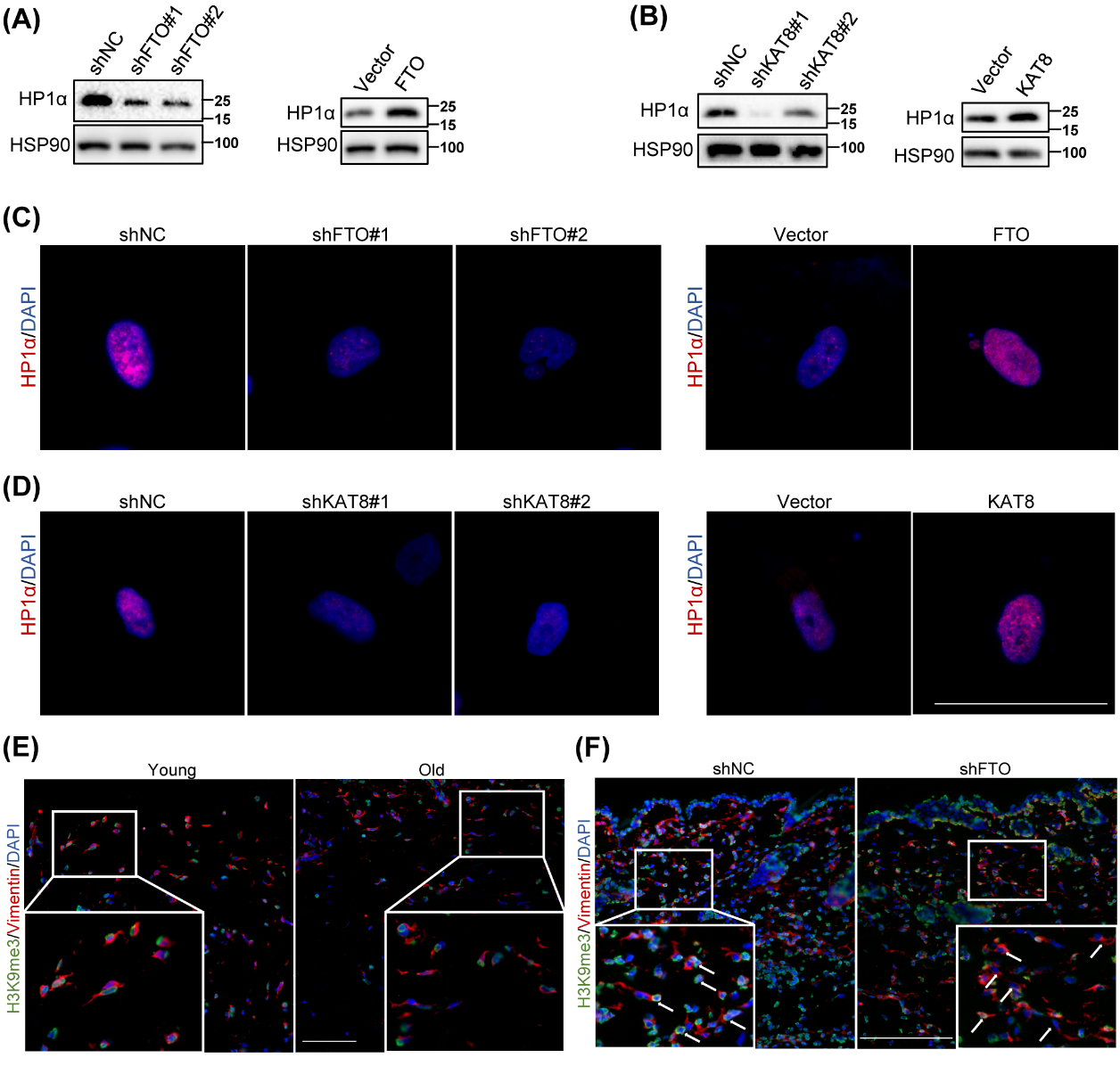


**Figure S10.**

Expression of heterochromatin in HDFs or skin tissues after different treatments.

(A and C), The protein levels of HP1α in HDFs upon knockdown or overexpression of FTO detected by western blotting (A) and immunofluorescence staining (C). (B and D), The protein levels of HP1α in HDFs upon knockdown or overexpression of KAT8 by by western blotting (B) and immunofluorescence staining (D). (E and F), The levels of H3K9me3 in young or old human skin tissues (E) and control group or FTO-depleted skin tissues (F) by immunofluorescence staining. White arrows indicate fibroblasts. Data are representative of at least three independent experiments. Scale bar, 100 μm. Data are shown as mean ± SEM. *P < 0.05; **P < 0.01; ***P < 0.001; ****P < 0.0001; ns, not significant.


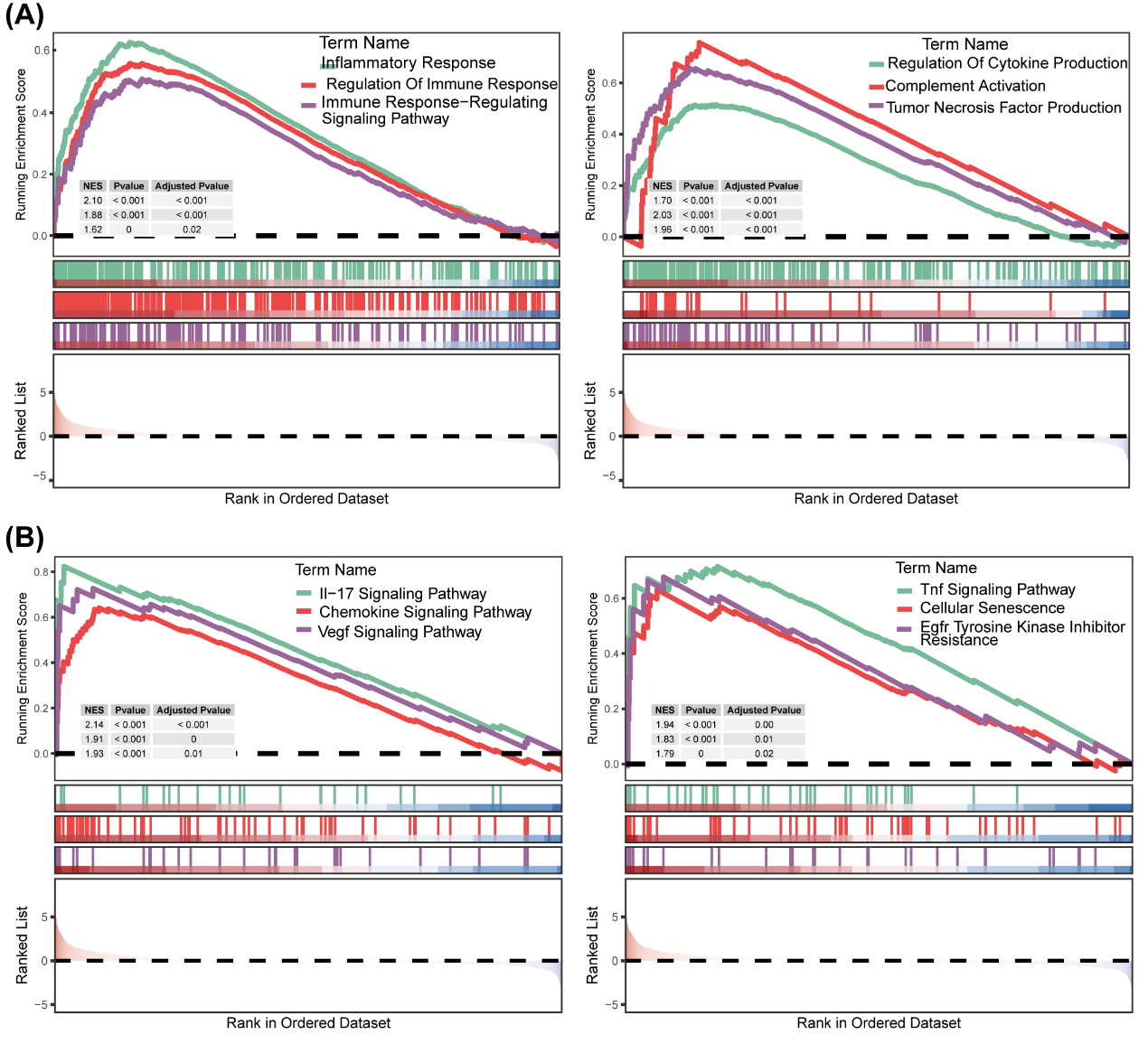


**Figure S11.**

Proteomic Data Analysis of FTO-depleted skin tissues.

(A and B), GSEA-GO analysis (A) or GSEA-KEGG analysis (B) illustrated the enrichment of biological processes or pathways of FTO-depleted skin tissues of wild-type C57BL/6 mice.
